# Single-cell ultra-high-throughput multiplexed chromatin and RNA profiling reveals gene regulatory dynamics

**DOI:** 10.1038/s41592-025-02700-8

**Published:** 2025-05-26

**Authors:** Sara Lobato-Moreno, Umut Yildiz, Annique Claringbould, Nila H. Servaas, Evi P. Vlachou, Christian Arnold, Hanke Gwendolyn Bauersachs, Víctor Campos-Fornés, Minyoung Kim, Ivan Berest, Karin D. Prummel, Kyung-Min Noh, Mikael Marttinen, Judith B. Zaugg

**Affiliations:** 1 European Molecular Biology Laboratory, Molecular Systems Biology Unit, Heidelberg, Germany; 2 European Molecular Biology Laboratory, Genome Biology Unit, Heidelberg, Germany; 3 Faculty of Biosciences, Collaboration for Joint PhD Degree between EMBL and Heidelberg University, Heidelberg, Germany; 4 Department of Internal Medicine, Erasmus Medical Centre Rotterdam, Rotterdam, the Netherlands; 5 Molecular Medicine Partnership Unit, Heidelberg, Germany; 6 Department of Biomedicine, Aarhus University, Aarhus, Denmark; 7 Faculty of Medicine and Health Technology, Tampere University, Tampere, Finland; 8 Department of Biomedicine, University of Basel, Basel University Hospital, Basel, Switzerland

**Keywords:** Molecular biology, Biological techniques

## Abstract

Enhancers and transcription factors (TFs) are crucial in regulating cellular processes. Current multiomic technologies to study these elements in gene regulatory mechanisms lack multiplexing capability and scalability. Here we present single-cell ultra-high-throughput multiplexed sequencing (SUM-seq) for co-assaying chromatin accessibility and gene expression in single nuclei. SUM-seq enables profiling hundreds of samples at the million cell scale and outperforms current high-throughput single-cell methods. We demonstrate the capability of SUM-seq to (1) resolve temporal gene regulation of macrophage M1 and M2 polarization to bridge TF regulatory networks and immune disease genetic variants, (2) define the regulatory landscape of primary T helper cell subsets and (3) dissect the effect of perturbing lineage TFs via arrayed CRISPR screens in spontaneously differentiating human induced pluripotent stem cells. SUM-seq offers a cost-effective, scalable solution for ultra-high-throughput single-cell multiomic sequencing, accelerating the unraveling of complex gene regulatory networks in cell differentiation, responses to perturbations and disease studies.

## Abstract (web summary)

This work presents SUM-seq, an ultra-high-throughput method for co-profiling chromatin accessibility and gene expression in single nuclei across multiplexed samples, advancing the study of gene regulation in diverse biological systems.

## Main

Most disease-associated genetic variants lay in noncoding regions (reviewed in ref. ^1^). At the same time, many diseases are characterized by perturbed gene regulatory processes driven by transcription factors (TFs), including imbalanced cellular differentiation. Therefore, to study such disease mechanisms requires a technology that allows the joint analysis of enhancers and gene-regulatory dynamics along a differentiation time course at single-cell resolution.

Advances in single-cell RNA sequencing (snRNA-seq) and single-nucleus assay for transposase-accessible chromatin sequencing (snATAC-seq) have revolutionized our understanding of cell states and responses to perturbations (reviewed in ref. ^2^). Particularly, multimodal profiling can unravel gene regulatory dynamics governing fundamental biological processes^3–5^. However, while scalability for individual modalities is available (for example, scifi-RNA-seq^6^, sci-RNA-seq^7^, dsciATAC-seq^8^ and sci-ATAC-seq^9^), the majority of current multiomic methods are limited in scalability, multiplexing capability or cost effectiveness (for example, 10x Multiome and ISSAAC-seq^10^), with a handful providing scalability in terms of number of cells assayed (for example, SHARE-seq^11^ and Paired-seq^12^) and multiplexing (for example, MultiPerturb-seq^13^), but at the expense of data complexity (Table 1).

**Table 1:** Comparison between SUM-seq and other ATAC and RNA single-cell methods

Method	Platform	Multiplexing capacity	Estimated cost	Throughput	Sensitivity	Public accessions
SUM-seq	Combination plate based and microfluidic	Yes, combination of plate-based and microfluidic indexing	<0.05€ per cell	Ultra-high; >50,000 profiles per microfluidic channel	High; average genes per cell: 2,058 (K562) Average fragments per cell: 5,164 (K562)	
10x Multiome	Microfluidic	No	~0.35€ per cell	High; ~10,000 profiles per microfluidic channel K562: 11,693 (ATAC), 13,228 (RNA)	High; average genes per cell: 2,515 Average fragments per cell: 9,258	K562 ATAC: ERR9847049 K562 RNA: ERR9847050
SNARE-seq2	Microfluidic	No	<0.1€ per cell	Low; K562: 198 (ATAC), 355 (RNA)	Low; average genes per cell: 361 Average fragments per cell: 595	K562 ATAC: SRR8528319 K562 RNA: SRR8528318
Paired-seq	Plate based	No	<0.05€ per cell	Ultra-high; (1.51 million nuclei barcoded; sublibrary sequenced); HEK293T: 1,357 (ATAC), 1,283 (RNA)	Low; average genes per cell: 536 Average fragments per cell: 1,506	HEK293T ATAC: SRR8980188 HEK293T RNA: SRR8980189
ISSAAC-seq	Microfluidic and plate based possible	No	~0.2€ per cell	High (microfluidic); K562: 5,700 (ATAC), 5,159 (RNA)	High; average genes per cell: 2,522 Average fragments per cell: 6,789	K562 ATAC: ERR9847051 K562 RNA: ERR9847052
Smart3-ATAC	Plate based	Theoretically possible, but limited in throughput	>0.25€ per cell	Low; time-course mouse gastrulation: 3,196 single sorted cells	High; average UMI^a^ per cell: 46,501 Average fragments per cell: N/A	N/A
SHARE-seq	Plate based	Yes, plate based	<0.05€ per cell	Ultra-high; K562: 5,219 (ATAC), 7,754 (RNA)	Low; average genes per cell: 241 Average fragments per cell: 352	K562 ATAC: SRR10428394 K562 RNA: SRR10428405
scCAT-seq	Plate based	Theoretically possible, but limited in throughput	>0.25€ per cell	Low; K562: 176 (ATAC), 74 (RNA)	High; average genes per cell: 10,173 Average fragments per cell: 212,991	SRP136421
MultiPerturb-seq	Microfluidic	Yes, combination of plate-based and microfluidic indexing	<0.05€ per cell	Not reported	Very low; average genes per cell: 83 Average fragments per cell: 681	PRJNA1160410

^a^Genes per cell not reported.

Here, we present single-cell ultra-high-throughput multiplexed sequencing (SUM-seq): a cost-effective and scalable sequencing technique for multiplexed RNA/ATAC profiling in single nuclei at ultra-high-throughput scale (up to millions of cells and hundreds of samples^6^). SUM-seq builds on the two-step combinatorial indexing approach, originally introduced by Datlinger et al.^6^ for snRNA-seq, extending it to the multiomic (RNA/ATAC) setup.

We demonstrate the application of SUM-seq in four experimental setups including species mixing, macrophage polarization, primary T cells and an arrayed CRISPR screen in induced pluripotent stem cells, profiling between 16 and 54 samples in each setup, including fixed and frozen samples. These applications exemplify the flexibility of SUM-seq to accommodate complex experimental setups in a cost-effective manner. We show how the multiomic readout can be used to infer enhancer-mediated gene regulatory networks (eGRNs), how it can resolve temporal patterns of gene regulation and how it enables interpretation of disease-associated genetic variants in noncoding regions. SUM-seq is compatible with fixed and frozen samples, thus ideal for projects requiring prolonged sample collection periods, such as time-course experiments or extensive atlas projects.

## Results

### Overview of the SUM-seq method

**Fig. 1:**
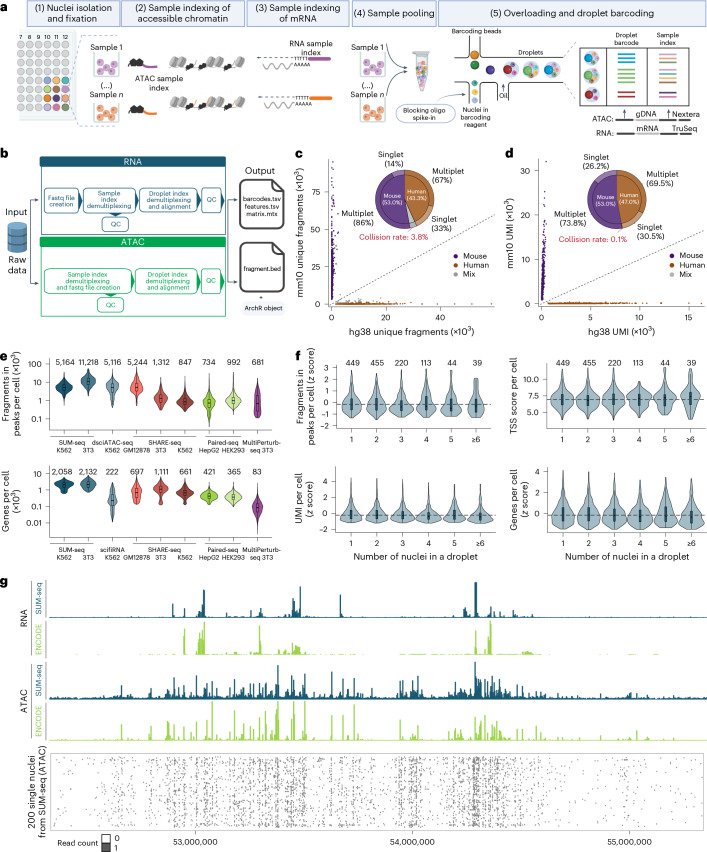
SUM-seq allows simultaneous profiling of chromatin accessibility and gene expression in single cells at ultra-high-throughput scale. **a**, A schematic of the SUM-seq workflow. The key steps and detailed structures are described in the main text and Extended Data Fig. 1. Created with BioRender.com. **b**, An overview of the computational analysis pipeline. **c**,**d**, Species-mixing plots for ATAC (**c**) and RNA (**d**) modalities, indicating the fraction of reads from singlets and multiplets assigned to the mouse (mm10, *y* axes) and human genome (hg38, *x* axes) with optimal assay parameters (fresh cells, including PEG and measures to mitigate Tn5 hopping). Genomic DNA fragments (**c**) and transcripts (**d**) are demultiplexed with the sample index and the droplet barcode. The pie chart shows the fraction of human and mouse cells and the frequency of multiplets and singlets. Collision rates are highlighted in red. **e**, The number of accessible DNA fragments in peaks (FiPs; top) and genes (bottom) per cell for SUM-seq (*n* = 699 (3T3), 621 (K562) cells) and other methods (dsciATAC-seq^8^: GSM3507387, *n* = 1,773 cells; SHARE-seq^11^: GSM4156590, GSM4156594 and GSM4156596 (ATAC), *n* = 1,216 (GM12878), 2,044 (3T3) and 4,984 (K562) cells; GSM4156602, GSM4156603, GSM4156605 and GSM4156607 (RNA), *n* = 2,4743 (GM12878), 2,637 (3T3) and 6,025 (K562) cells; Paired-seq^12^: GSM3737488 (ATAC), *n* = 690 (HepG2) and 862 (HEK293) cells; GSM3737489 (RNA), *n* = 629 (HepG2) and 643 (HEK293) cells; scifi-RNA-seq^6^: GSM5151362, *n* = 1,606 cells; MultiPerturb-seq^13^: GSM8528725, *n* = 12,490 (ATAC) and 921 (RNA) cells). The median is indicated above each violin plot. **f**, Distribution of FiPs and TSS scores per cell (top) as well as UMIs and genes per cell (bottom), split by the number of nuclei in a droplet (*n* represents the number of droplets containing the indicated number of nuclei and is shown above each violin plot). **g**, Top: a representative genome browser view (chr12: 52,334,173–55,349,410) of SUM-seq data is shown for K562 as aggregated transcript and ATAC fragment mappings. K562 ATAC-seq and RNA-seq data tracks were retrieved from the ENCODE database (tracks labeled in green; GSE86660^19^ (RNA) and GSE170214^19^ (ATAC)). Bottom: Binarized accessibility for 200 randomly selected single-nucleus profiles. In the box plots in **e** and **f**, the center line represents the median, and the lower and upper hinges the 25th and 75th quartiles, respectively. The whiskers represent values that fall within 1.5 × the interquartile range between the 25th and 75th percentiles.

**Extended Data Fig. 1:**
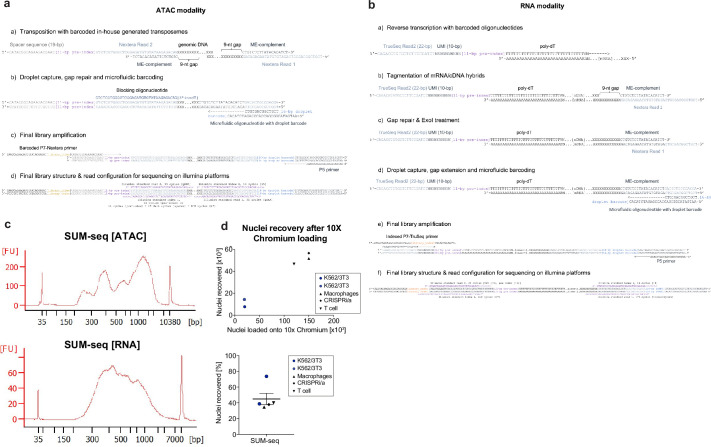
Detailed library structures of SUM-seq and performance metrics. **a**, Detailed outline with oligonucleotide sequences of the ATAC modality of SUM-seq for each step of the experimental workflow. **b**, Detailed schematic outline with oligonucleotide sequences of the RNA modality of SUM-seq. **c**, Bioanalyzer profiles of final SUM-seq snATAC- (upper panel) and snRNA-seq (lower panel) libraries. **d**, Raw recovery values (upper panel, x-axis depicts number of nuclei loaded to the microfluidic lane and y-axis the number of nuclei in the final QC filtered data matrix) and percentages (lower panel, (number of nuclei loaded to the microfluidic channel / number of nuclei in the final QC filtered data matrix) x 100) of nuclei recovery before and after 10x Chromium loading. Scatter plot shows individual values with the mean value ± s.e.m. (standard error; *n* = 5 biological replicates).

SUM-seq combines the concepts of combinatorial fluidic indexing (for example, scifi-RNA-seq^6^) and the single-plex multiomic assay ISSAAC-seq^10^, enabling single-cell RNA/ATAC profiling of hundreds of samples and 1.5 million cells in one 10x Chromium channel. First, nuclei are isolated, fixed with glyoxal and distributed into equal bulk aliquots. In steps two and three, unique sample indices are introduced for the ATAC and RNA modalities. For ATAC, accessible genomic regions are indexed by Tn5 loaded with barcoded oligos. For RNA, the mRNA molecules are indexed with barcoded oligo-dT primers via reverse transcription (RT) (Fig. 1a, Extended Data Fig. 1a,b and Supplementary Table 1). In step four, samples are pooled for tagmentation of cDNA–mRNA hybrids to introduce a primer binding site necessary for the second microfluidic barcoding. In step five, nuclei are overloaded onto a microfluidic system (for example, 10x Chromium), resulting in multiple nuclei per droplet. Within these droplets, the fragments receive a second barcode (droplet barcode). This dual barcoding allows assignment of sequencing reads to individual nuclei even if they were within the same droplet. In step 6, droplets are broken, both modalities pre-amplified and the library split into two equal proportions for further modality-specific amplification. At this stage, a library index can be introduced for multiplexing libraries for sequencing. For SUM-seq data processing, we have developed a scalable and reproducible Snakemake pipeline^14^, in which reads are assigned to sample indices and demultiplexed to single-cell resolution by droplet barcodes (Fig. 1b). From here, reads are mapped and a gene expression matrix and tile matrix are generated for RNA and ATAC, respectively. Modalities are matched based on the assigned sample index–droplet barcode combinations.

### SUM-seq enables scalable single-cell RNA/ATAC profiling

**Extended Data Fig. 2:**
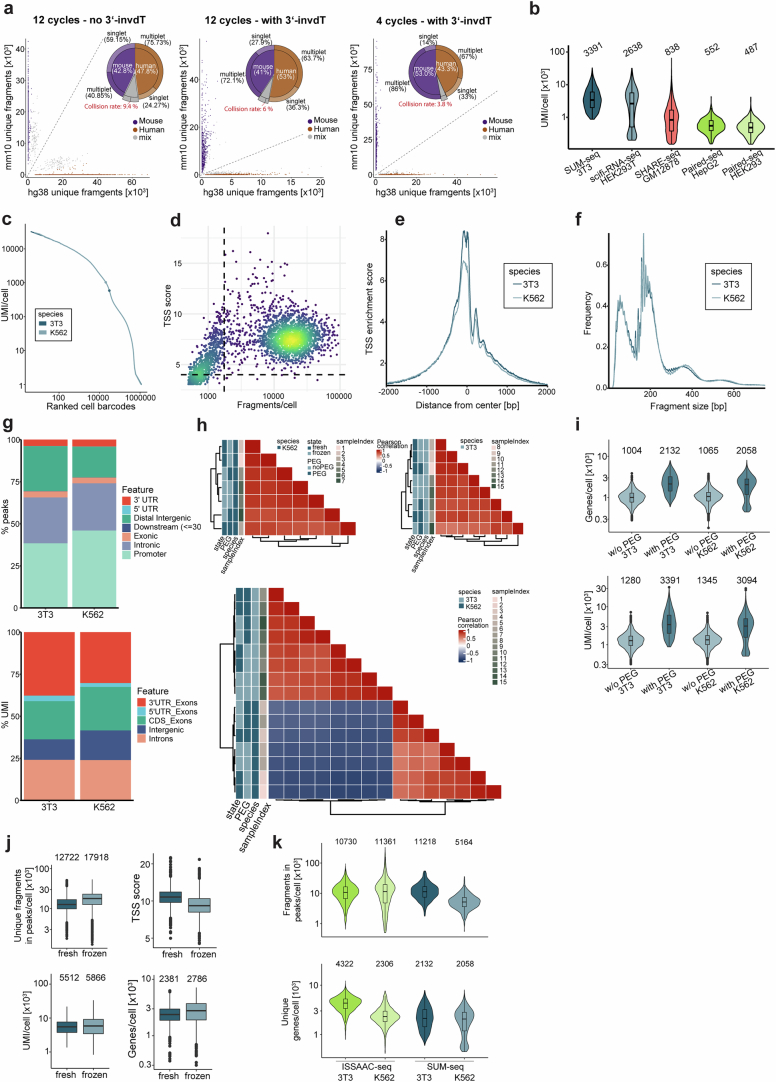
Performance metrics of SUM-seq and optimization of experimental parameters. **a**, Impact of using the blocking oligonucleotide and reducing thermal amplification cycles on collision rates (highlighted in red) as a function of mapped reads to mouse (mm10, y-axes) and human (hg38, x-axes) genomes. Pie chart shows the fraction of human and mouse cells as well as the multiplet and singlet frequency. **b**, UMIs detected per cell for SUM-seq, scifi-RNA-seq, SHARE-seq and Paired-seq in the indicated cell lines (datasets indicated in Fig. 1e (lower panel)). **c**, Ranked number of UMIs (y-axis) per cell. Inflection points are indicated as points along the curve**. d**, TSS score (y-axis) and number of fragments (x-axis) per cell for the ATAC modality of NIH-3T3s. Dashed lines indicate thresholds for cell calling. **e**, Enrichment of ATAC-reads (y-axis) displayed around the TSS ( ± 2 kb). Light blue: K562, dark blue: NIH-3T3. **f**, Size distribution of ATAC fragments in the indicated cell lines in base pairs (x-axis). **g**, Genomic annotation of mapped ATAC fragments (upper panel) and transcripts (lower panel) in K562 and NIH-3T3 cells**. h**, Correlation heatmaps of sample indices for the ATAC- (upper panels) and RNA-modalities (lower panel) of the species-mixing experiment. Rows are annotated according to their state (fresh or frozen), cell type (K562, NIH-3T3), inclusion of PEG during RT and the sample index. **i**, Violin plots displaying the effect of PEG on the number of genes (upper panel) and UMIs (lower panel) detected in the RNA modality of SUM-seq in NIH-3T3 and K562 cells. 3T3: w/o PEG, n = 2428; with PEG, n = 699. K562: w/o PEG, n = 2842; with PEG, n = 621 cells. **j**, Impact of cryopreservation on the data complexity. The upper panel displays key quality metrics comparison for the ATAC modality (fragments in peaks per cell and TSS enrichment score), and the lower panel for the RNA modality (genes and UMIs per cell). ATAC (top panel): fresh, n = 2499; frozen, n = 1566, RNA (bottom panel): fresh, n = 2476; frozen, n = 1399 cells. **k**, Comparison of quality metrics of ISSAAC-seq and SUM-seq in K562 and NIH-3T3 cells (top: fragments in peaks per cell, bottom: genes per cell). ISSAAC-seq metrics retrieved from https://github.com/dbrg77/ISSAAC-seq/tree/main. SUM-seq: n = 699 (3T3), n = 621 (K562). ISSAAC-seq: ATAC; n = 3032 (3T3), 5702 (K562). RNA; n = 3011 (3T3), 5161 (K562). Boxplots in **b**, **i**, **j**, **k**: Center line represents median; lower and upper hinges represent the 25th and 75th quartiles respectively. The whiskers represent values that fall within 1.5 times the interquartile range between the 25th and 75th percentiles.

To evaluate and optimize data quality, we performed SUM-seq on an equal mixture of human leukemia (K562) and mouse fibroblast (NIH-3T3) cells, using 16 sample indices (Methods and Extended Data Fig. 2a,h–k). This resulted in 100,000 dual-barcoded nuclei that we loaded into a single 10x Chromium channel (~7-fold overloading compared with the standard workflow). Note, to limit sequencing costs, we processed only 20% (20,000) of the droplets for final library preparation. This resulted in 6,215 human and 7,607 mouse cells with data from both modalities (~70% recovery of 10x Chromium input), representing a ~7-fold increase in throughput compared with the standard workflow (Extended Data Fig. 1d). Using this setup, we found that adding polyethylene glycol (PEG) to the RT reaction increased the number of unique molecular identifiers (UMIs) and genes detected per cell (~2.5- and ~2-fold), consistent with previous studies^15,16^ and with minor impact on ATAC quality (Extended Data Fig. 2h,i).

In the first experiments, we observed barcode hopping within multinucleated droplets^17,18^, primarily affecting the ATAC modality. To mitigate this, we developed two complementary strategies: (1) adding a blocking oligonucleotide^18^ in excess to the droplet barcoding step and (2) reducing the number of linear amplification cycles during droplet barcoding from 12 to 4 (Methods and Extended Data Fig. 2a). This resulted in human and mouse reads being well-separated, with a collision rate of 0.1% (UMIs) and 3.8% (ATAC fragments) (Fig. 1c,d). Finally, we found that glycerol-based cryopreservation after glyoxal fixation had minimal impact on the performance metrics of the assay (Extended Data Fig. 2j). Thus, SUM-seq supports asynchronous sampling by enabling sample gathering, fixation and cryopreservation before library preparation.

Performance metrics of snRNA (UMIs and genes per cell) and snATAC (fragments in peaks per cell, transcription start site (TSS) enrichment score and fragment size distribution) were consistently high for both modalities in SUM-seq, and library complexity metrics (genes and fragments in peaks per cell) outperformed other ultra-high-throughput assays for scRNA-seq, snATAC and multiomic approaches (Fig. 1e, Table 1 and Extended Data Fig. 2c–g). Compared with the single-plex assay ISSAAC-seq, we observed similar performance with a slight reduction in complexity (Extended Data Fig. 2k). Notably, the data from nuclei in overloaded droplets maintained the same quality as those from single-nuclei droplets (Fig. 1f). Importantly, ~90% of mapped ATAC fragments and 70% of RNA UMIs were assigned to cells, indicating low ambient material. Last, the aggregate of snRNA and snATAC data resembled the published bulk RNA-seq and ATAC-seq in the K562 benchmarks from ENCODE data^19^ (Fig. 1g).

### SUM-seq captures dynamics of macrophage polarization

Next, we used SUM-seq to study gene expression and TF activity during macrophage polarization. Macrophages are innate immune cells that can polarize toward a proinflammatory M1 or an anti-inflammatory M2 state depending on microenvironmental signaling. While key TFs for M1 and M2 polarization have been identified previously^20–22^, the complex regulatory networks orchestrating macrophage polarization over time remain elusive.

**Fig. 2:**
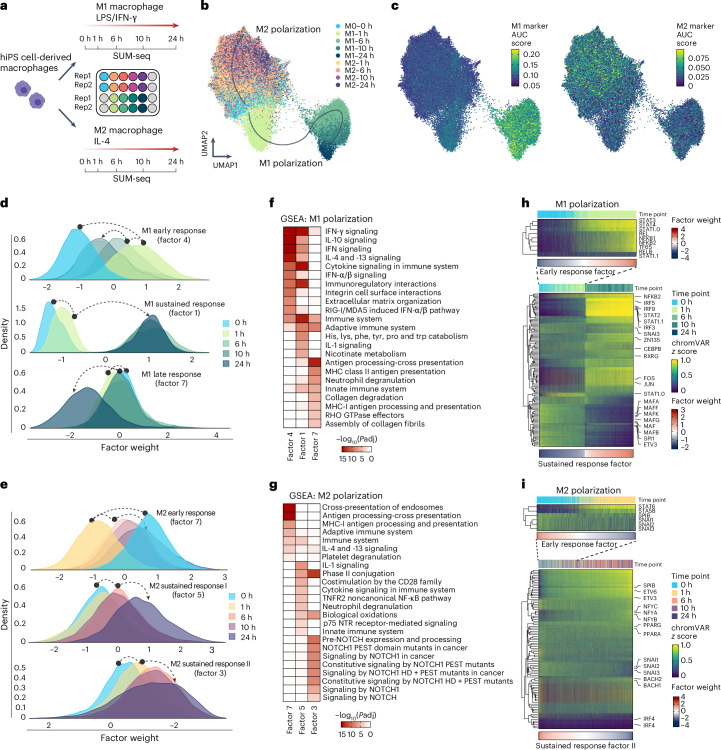
Integrated chromatin accessibility and gene expression data at single-nucleus resolution via SUM-seq characterize differentiation trajectories along M1/M2 macrophage polarizations. **a**, A schematic overview of the macrophage polarization experiment. hiPS cell-derived macrophages were stimulated with LPS and IFN-γ (M1) or IL-4 (M2). Nuclei were fixed with glyoxal and collected for SUM-seq at 0 h, 1 h, 6 h, 10 h and 24 h (*n* = 2 per time point). **b**,**c**, WNN UMAP projection of integrated SUM-seq data of macrophage polarization. Cells are annotated and labeled according to their sample index (arrows were drawn by hand for ease of interpretation) (**b**), and annotated with AUC score of M1 signature genes (**c**, left) and M2 signature genes (**c**, right; Supplementary Table 2). **d**,**e**, Distributions of cells from each time point across three MOFA factor weights associated with M1 polarization (**d**) (early response, sustained response and late response) and M2 polarization (**e**) (early response and sustained responses I and II). The dashed arrows indicate the direction of the time-resolved response. **f**,**g**, GSEA (Methods) for M1 (**f**) and M2 (FDR-corrected *P* values (*P*adj)) (**g**) polarization factors are shown as heat maps. **h**, Top: motif activity (rank-normalized chromVAR *z* scores) for TFs associated with M1 early response (Methods) across M0 and M1–1 h cells sorted by M1 early response factor weights. Bottom: motif activity for TFs associated with M1 sustained response across all M0 and M1 cells sorted by sustained factor weights. STAT1.0 and STAT1.1 represent the homodimer and ISGF3 motifs of STAT1, respectively. **i**, Top: motif activity for TFs associated with M2 early response across M0 and M2–1 h cells sorted by M2 early response factor weights. Bottom: motif activity for TFs associated with M2 sustained response II across all M0 and M2 cells sorted by sustained factor II weights.

**Extended Data Fig. 3:**
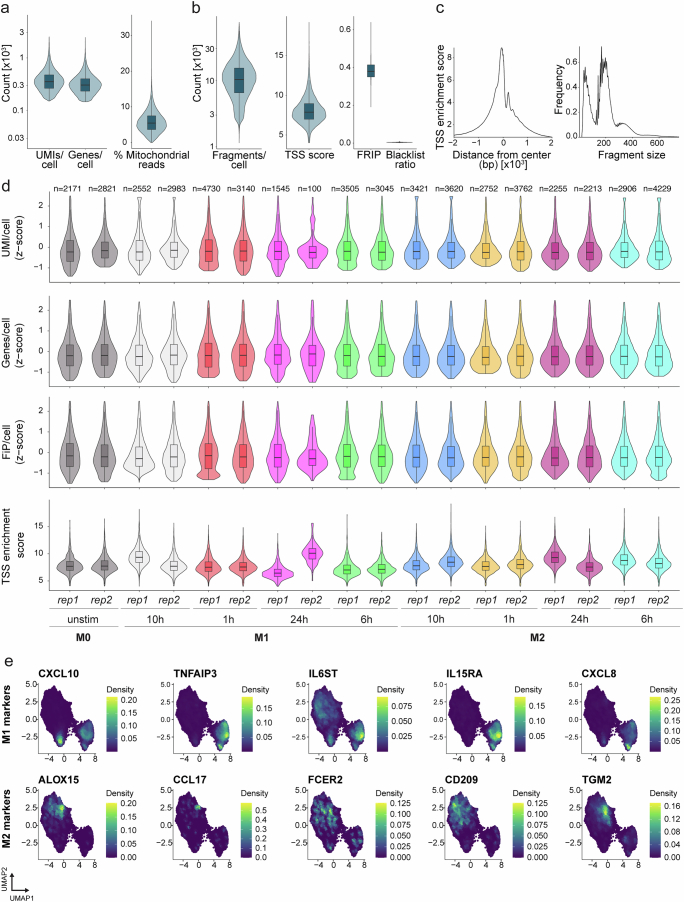
Quality control metrics of M1/M2 polarization SUM-seq experiment. **a-b**, Violin plots showing the number of UMIs, genes and percentage of mitochondrial reads per cell captured in the RNA modality (**a**) and unique fragments, TSS enrichment score, and fraction of reads in peaks (FRIP) per cell captured in the ATAC modality (**b**) of the M1/M2 polarization SUM-seq experiment. **c**, Enrichment of ATAC-reads (y-axis) displayed around the TSS ( ± 2 kb) (left). Size distribution of ATAC fragments in the indicated cell lines in base pairs (x-axis) (right). **d**, Violin plots showing z-score-transformed quality control metrics for RNA (UMIs per cell, genes per cell) and ATAC (FiP; Fragments in Peaks per cell, TSS enrichment per cell) in the macrophage polarization SUM-seq experiment across replicates and conditions. n indicates the number of cells per condition and is shown above the violin plots. **e**, Weighted nearest neighbor (WNN) UMAP projection of integrated SUM-seq data from macrophage polarization, showing the weighted kernel density estimation for expression of M1- and M2-specific marker genes. Boxplots in **a**, **b**, **d**: Center line represents median; lower and upper hinges represent the 25th and 75th quartiles respectively. The whiskers represent values that fall within 1.5 times the interquartile range between the 25th and 75th percentiles.

We profiled human induced pluripotent stem (hiPS) cell-derived macrophages across a polarization time course from the naive M0 state to M1 (with lipopolysaccharide (LPS) and IFN-γ) and M2 states (with IL-4) using SUM-seq. We collected samples in duplicates at five time points along the two polarization trajectories: before (M0) and 1, 6, 10 and 24 h after stimulation (Fig. 2a). We loaded 150,000 nuclei of these 18 samples into a single 10x Chromium channel, resulting in multiomic data for 51,750 nuclei passing quality control (QC) filtering (Methods) that were evenly distributed across samples (Fig. 2b and Extended Data Fig. 3). For snATAC, post-QC nuclei had 11,900 unique fragments on average, a TSS score of 8 and 40% reads in peaks (with 45,000 reads per cell). For snRNA-seq, nuclei displayed on average 407 UMIs and 342 genes (with 35,000 reads per cell; Extended Data Fig. 3a–c). No difference in these metrics was observed between samples (Extended Data Fig. 3d). We note that the omission of PEG during RT (owing to technical reasons; Methods) led to the relatively lower UMI and gene counts per nucleus compared with the mixed-species experiment. Despite the lower average UMI counts, the high number of nuclei that passed the quality filter enabled effective downstream analyses.

The joint uniform manifold approximation and projection (UMAP) visualization revealed separation of cells along the M1 and M2 polarization trajectories (Fig. 2b), both exhibiting specific expression of well-established markers (Extended Data Fig. 3e). Further, we observed increased expression of literature-derived M1 and M2 module scores (Supplementary Table 2 and Methods) along their respective polarization trajectories (Fig. 2c), confirming the validity of our experimental setup.

**Extended Data Fig. 4:**
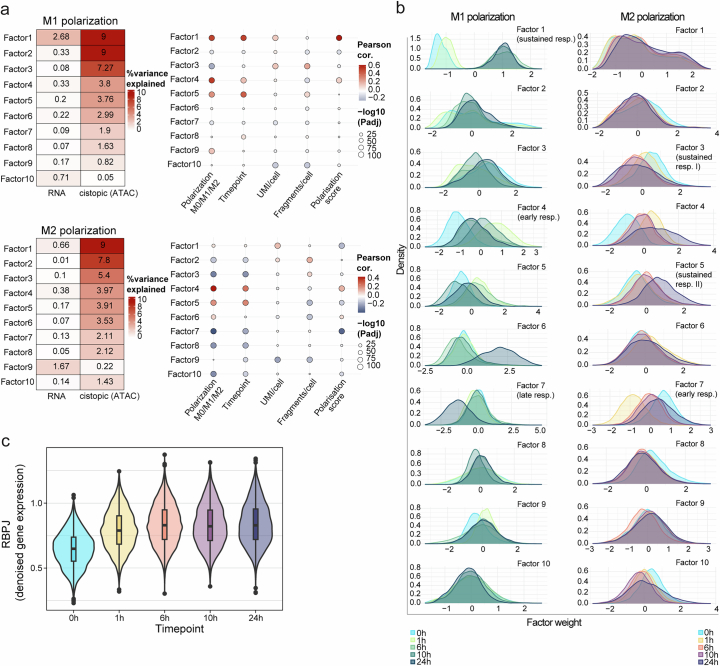
Relations of M1/M2 polarization latent factors with metadata. **a**, Left: Percentage of variance explained by each MOFA-inferred latent factor in RNA and ATAC modalities for M0/M1 cells (top) and M0/M2 cells (bottom). Right: Correlation between latent factors and metadata, represented by dot plots for M0/M1 cells (top) and M0/M2 cells (bottom). Dot color indicates the Pearson correlation coefficient, and dot size reflects statistical significance (FDR corrected p-value). **b**, Distributions of cells from each time point across MOFA factor weights associated with M1 polarization (left) and M2 polarization (right). **c**, MOFA denoised gene expression of RBP-J per timepoint for M0 and M2 polarized cells. Center line of boxplots represent median; lower and upper hinges represent the 25th and 75th quartiles respectively. Whiskers represent values that fall within 1.5 times the interquartile range between the 25th and 75th percentiles.

To delineate the key features that influence gene expression and chromatin accessibility during macrophage M1/M2 polarization, we performed multiomics factor analysis (MOFA)^23^ (Extended Data Fig. 4a,b). Three MOFA factors were associated with M1 polarization, explaining the early response (factor 4), sustained response (factor 1) and late response (factor 7) (Fig. 2d and Extended Data Fig. 4a). For M2 polarization, we identified an early response (factor 7) and two distinct sustained response factors (factors 3 and 5) (Fig. 2e).

Genes associated with the early M1 response (factor 4) were enriched in IFN-γ and cytokine signaling (Fig. 2f, Methods and Supplementary Table 3). Genes associated with the sustained response (factor 1), were also enriched in IFN signaling along with general immune system processes, IL-1 signaling and metabolic terms, in line with metabolic reprogramming previously observed in M1 macrophages^24^. The M1 late response genes were predominantly enriched for antigen (cross-) presentation and RHO signaling (Fig. 2f).

The early M2 polarization factor (factor 7) was enriched for IL-4 signaling, while the two M2 sustained factors (factors 3 and 5) were enriched in biological oxidation—probably signifying the switch in metabolism of M2 macrophages^25^, and noncanonical NF-κB signaling and Notch signaling (Fig. 2g). While Notch signaling is commonly associated with M1 polarization, it also plays a context-dependent role in M2 polarization; for example, RBP-J, a key regulator of Notch signaling was associated with factor 3 and upregulated in M2 macrophages over time (Extended Data Fig. 4c), which aligns with its role in promoting M2 polarization in specific inflammatory contexts^26,27^.

### SUM-seq uncovers TF activity dynamics of M1/M2 polarization

**Extended Data Fig. 5:**
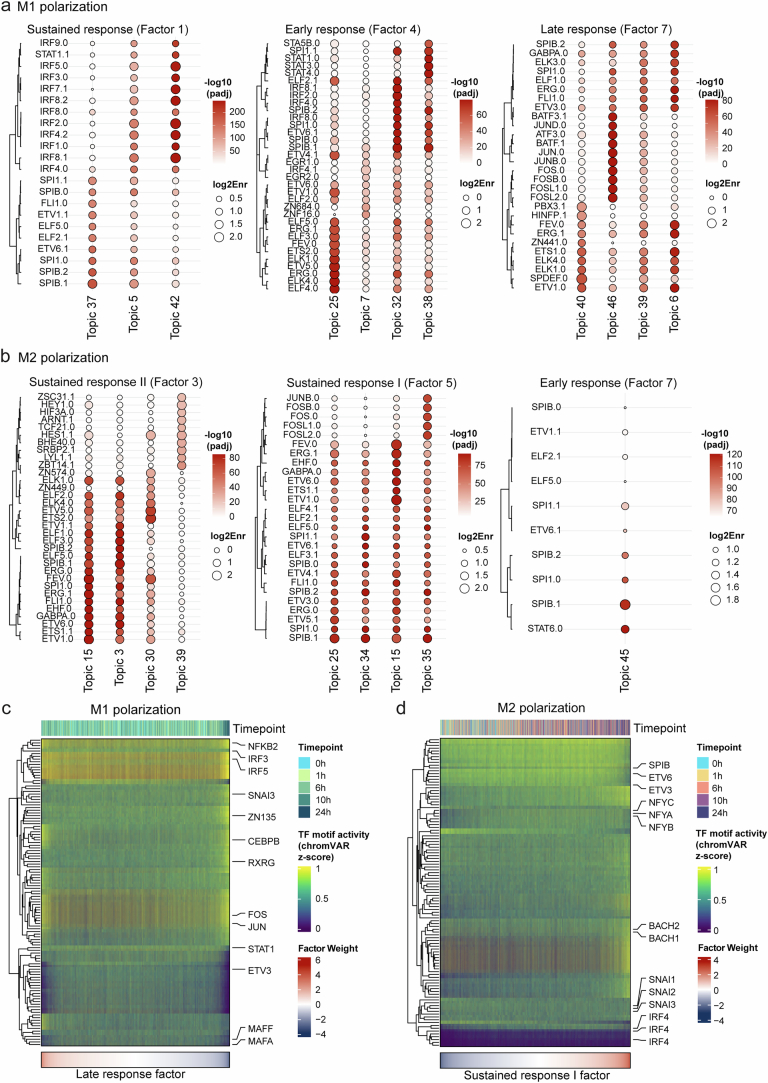
Motif enrichment for peaksets associated with M1/M2 latent factors and TF motif activity for M1 late response- and M2 sustained response II TFs. **a**, Top 10 TF motifs enriched per topic peaksets associated with M1 sustained response (left), M1 immediate response (center) and M1 late response (right) factors. **b**, Top 10 TF motifs enriched per topic peaksets associated with M2 sustained response I (left), M2 sustained response II (center) and M2 immediate response (right) factors. Dot size indicates log2 enrichment and color -log10(FDR corrected p-values). **c**, Motif activity (rank normalized chromVAR z-scores) for TFs associated with M1 late response (Methods) across all M0 and M1 cells sorted by M1 late response factor weights **d**, Motif activity for TFs associated with M2 sustained response II across all M0 and M2 cells sorted by M2 sustained response II factor weights.

Next, we investigated which TFs underlie chromatin remodeling during M1 polarization. Using chromVAR, we quantified TF motif accessibility variation^28^ as a proxy for a TF binding to a particular motif (TF motif activity). We selected the top 10% most variable TF motifs and filtered for those enriched in peak sets associated with either the early, sustained or late response of M1 (Extended Data Fig. 5a), resulting in 103 unique TFs (139 motifs; Supplementary Table 4).

Motif activity of prototypical M1 TFs, such as STAT1 and IRF5 (refs. ^21,22^), increased along the M1 early response (factor 4; only M0 and M1–1 h cells), followed by activation of NF-κB (NFKB1, NFKB2, RELA, REL and TF65; Fig. 2h). Furthermore, the AP-1 complex motifs (JUN, FOS and so on) were transiently upregulated, indicative of a cellular activation state^29^. Meanwhile, the myeloid differentiation factors of the ETS family, including SPI1 (PU.1)^30^, were active in M0 macrophages but showed a slight decrease in activity upon polarization.

Along the sustained M1 response (factor 1), we observed a switch-like increase in motif activity for many prototypical M1 TFs, including STATs and IRFs, such as IRF5 (Fig. 2h). A notable observation was STAT1, which functions either as a homodimer (also called IFN-γ activated factor (GAF)) or as part of the interferon-stimulated gene factor-3 (ISGF3) complex with STAT2 and IRF9 (ref. ^31^). The HOCOMOCO TF motif database contains two STAT1 motifs: STAT1.H12INVIVO.0.P.B (STAT1.0), bound by the STAT1 homodimer, and STAT1.H12INVIVO.1.P.B (STAT1.1), which resembles the STAT2 motif^32^ and probably corresponds to the ISGF3-bound motif. After an initial rise, STAT1 homodimer motif activity steadily dropped along the sustained response, while the ISGF3 motif activities (STAT1.1, STAT2 and IRF9) increased (Fig. 2h). This highlights the role of the STAT1 homodimer in initiating chromatin remodeling in response to IFN-γ, which is later replaced by the ISGF3 complex driving the type-I IFN response^31^.

During the late response (factor 7), TF motif activity remained stable, with only slight increases in NFY motifs and a slight decrease in CEBP motifs (Extended Data Fig. 5c), suggesting little TF-driven gene regulation between 6 and 24 h of M1 polarization.

A similar analysis of the most variable 93 TFs (121 motifs) along the M2 polarization (Extended Data Fig. 5b and Supplementary Table 4) revealed a less striking dynamic. Most notable, we observed increasing motif activity for the IL-4-responsive STAT6 (ref. ^33^) motif along the early M2 response (Fig. 2i) and a steady increase in ETS motif activity (SPIB^34^, ETV3, ETV6 (ref. ^35^) and NFY) along the sustained response (Fig. 2i and Extended Data Fig. 5d). Conversely, motif activity of SNAIL and CTCF, a protein shaping chromatin structure^36^, decreased along the sustained response, the latter suggesting a global rewiring of chromatin architecture.

Overall, we observed strong remodeling of the chromatin landscape along the macrophage polarization trajectories, much of it driven by specific sets of TFs.

### GRN analysis reveals sustained response by ISGF3

**Fig. 3:**
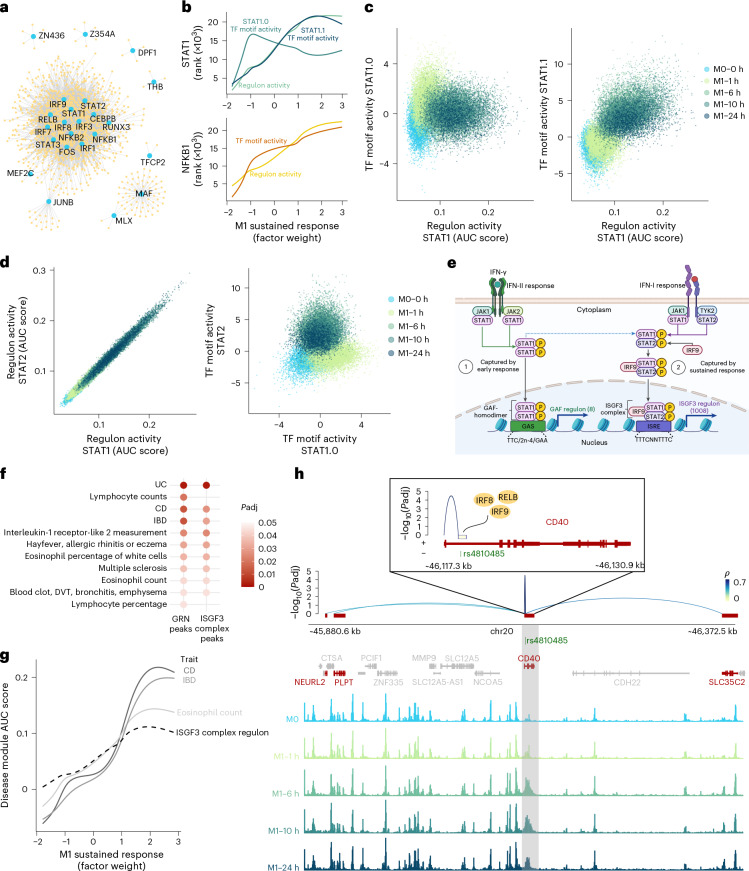
GRNs inferred from SUM-seq data in macrophages coupled with genetic evidence reveal TF and regulon hierarchy, linking TFs to immune traits. **a**, GRN visualization of TFs and their assigned target genes, including TFs with ≥4 targets. **b**, TF motif activity (rank chromVAR *z* score) and regulon activity (rank AUC cell score) for STAT1 (top) and NFKB1 (bottom) motifs along the M1 sustained response factor. Cells are ranked by their respective value for activity measures; the lines show best fit (generalized additive model). **c**, Scatter plots of the correlation between the STAT1 regulon activity and STAT1.0 (left) and STAT1.1 (right) TF motif activities. The points represent cells and are colored by time point. **d**, Scatter plots of each cell across the M1 macrophage polarization showing the correlation between STAT1 and STAT2 regulons (left) and STAT1.0 and STAT2 TF motif activity (right). The points represent cells and are colored by time point. **e**, A schematic of the type II IFN-associated STAT1 homodimer (GAF) and the type I IFN-associated STAT1/STAT2/IRF9 (ISGF3 complex) responses (as reviewed in ref. ^81^). The type II response is represented by the early response (indicated as 1; GAF regulon), and the type I response by the sustained response (indicated as 2; ISGF3 regulon). Regulon sizes are in brackets. Created with BioRender.com. **f**, Diseases and traits associated with genetic variants enriched in open regions of all GRN peaks and a highly connected regulon in the eGRN (the STAT1/STAT2/IRF9: putative ISGF3 complex) using LDSC (FDR-corrected *P* values). All open regions in macrophages are used as background. DVT, deep vein thrombosis. **g**, AUC cell scores for disease modules derived by intersecting putative genome-wide SNPs for the top enriched diseases with the putative ISGF3 complex regulon (Methods) compared with AUC cell scores for all ISGF3 regulon genes along the M1 sustained response factor. **h**, Top: eGRN peak gene interactions for the CD40 intronic peak intersecting IBD-associated SNP rs4810485, zooming in to the CD40 region with the TFs binding to this peak (inset). Bottom: M0 and M1 cell aggregate ATAC-seq tracks split by time point highlighting the CD40 intronic peak. Chr, chromosome.

**Extended Data Fig. 6:**
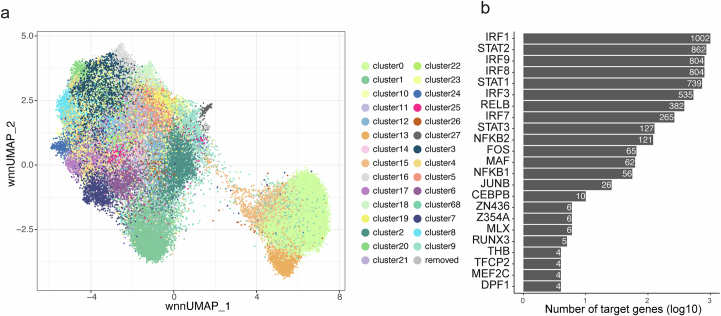
Cluster-based pseudobulking strategy and transcription factor connectivity of the macrophage polarization eGRN. **a**, Weighted nearest neighbor (WNN) UMAP projection of integrated SUM-seq data from macrophage polarization, colored by the cell clusters used for pseudobulking and subsequently inferring an eGRN. Only clusters with more than 25 cells were included to make the eGRN. **b**, Degree plot of number of target genes linked to each transcription factor (TF) in the eGRN. Only TFs with at least 4 connected genes are shown.

Next, we constructed an eGRN using GRaNIE^37^ (Methods and Supplementary Table 5) to study how TF-driven chromatin remodeling leads to activation of specific gene expression programs. The eGRN retrieved 44 TFs (out of the 103 associated with M1 polarization), either as a TF or as a target gene (Fig. 3a and Extended Data Fig. 6b). The most connected TFs shared many of their target genes (regulon). For example, the IRF1 (1,002 target genes) and IRF8 (804 target genes) regulons shared 728 genes.

**Extended Data Fig. 7:**
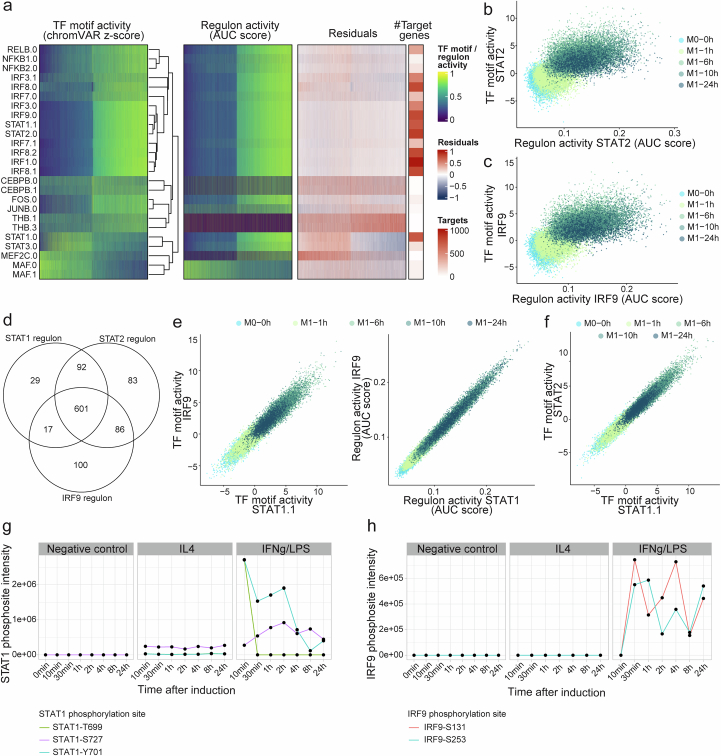
Dynamics of STAT1, STAT2, and IRF9 motif and regulon activity in M1 polarization. **a**, Heatmaps showing TF motif activity (rank normalized chromVAR z-score), regulon activity (rank normalized AUC score), and the similarity between the two, for M1 polarization-associated TFs found as TFs in the eGRN, across M0 and M1 cells sorted by the M1 sustained factor. **b**, Scatter plots of the correlation between the STAT2 regulon activity (AUC score) and TF motif activity (chromVAR Z-score) and **c**, IRF9 regulon activity and motif activity. Points represent cells and are coloured by their experimental time point. **d**, Venn diagram of STAT1, STAT2 and IRF9 regulon genes. **e**, Scatter plots of the correlation between STAT1.1 and IRF9 motif activities (top) and STAT1 and IRF9 regulon activities (bottom). Points represent cells and are coloured by their experimental time point. **f**, Scatter plots of the correlation between STAT1.1 and STAT2 motif activities (top) and STAT1 and STAT2 regulon activities (bottom). Points represent cells and are coloured by their experimental time point. **g**, STAT1 T699 and Y701 phosphopeptide intensities upon IFN-y/LPS stimulation of THP1 derived macrophages. **h**, IRF9 S131 and S253 phosphopeptide intensities upon IFN-y/LPS stimulation of THP1 derived macrophages.

We further resolved the hierarchy of a TF’s activity on chromatin accessibility and their effect on transcription by comparing motif and regulon activity similarity along the M1 immediate response factor (Methods). For most TFs, motif activity was positively correlated with the expression of their regulon (Extended Data Fig. 7a). One exception was NFKB1, whose regulon expression was slightly delayed relative to the gradual increase in its motif activity (Fig. 3b). Notably, STAT1 regulon activity was discordant with the activity of its homodimer (STAT1.0) motif, and instead correlated with ISGF3 (STAT1.1, STAT2 and IRF9) motif activities (Fig. 3b,c). In line with this, the STAT1.1, STAT2 and IRF9 regulons shared 601 target genes, whereas the STAT1.0 (homodimer) regulon contained only 8 genes (Fig. 3d, Extended Data Fig. 7b–f and Supplementary Table 6).

We hypothesize that the increased activity of STAT1.0 homodimer and other early response TFs after IFN-γ/LPS stimulation (Fig. 2h) leads to endogenous type-I IFN production, which in turn activates the ISGF3 complex, and may explain the delayed response of ISGF3 as compared to the STAT1.0 homodimer activity (Fig. 3e).

To support our findings, we assessed STAT1 and IRF9 phosphorylation, leveraging phosphoproteomic data from IFN-γ/LPS and IL-4-stimulated THP1-derived macrophages^38^. These data showed that STAT1 gets phosphorylated at T699 and Y701 immediately after IFN-γ/LPS stimulation, followed by a gradual decrease over time (Extended Data Fig. 7g). Phosphorylation of these sites is IFN induced and triggers STAT1 DNA binding^39,40^, in line with our data. Moreover, IRF9 phosphorylation at sites S131 and S253 show a delayed but sustained increase (Extended Data Fig. 7h), in line with the gradual increase in STAT2 and IRF9 motif activity in our data. S253 is induced by IFN-β (a type-I IFN) and may play a role in regulating the expression of interferon-stimulated genes and interactions with STAT2 (ref. ^41^).

SUM-seq revealed a marked shift in STAT1-mediated regulation, transitioning from its homodimer-driven chromatin remodeling during early M1 polarization to an ISGF3-driven response, reflected in both accessibility and transcription at later time points. This switch is in line with previous reports on the timing of IFN stimulation^42,43^.

### GWAS enrichment analyses of the SUM-seq identified eGRN

Next, we asked whether accessible chromatin regions linked to regulon genes in the network were enriched for heritability of specific diseases using linkage disequilibrium score regression (LDSC)^44^.

**Extended Data Fig. 8:**
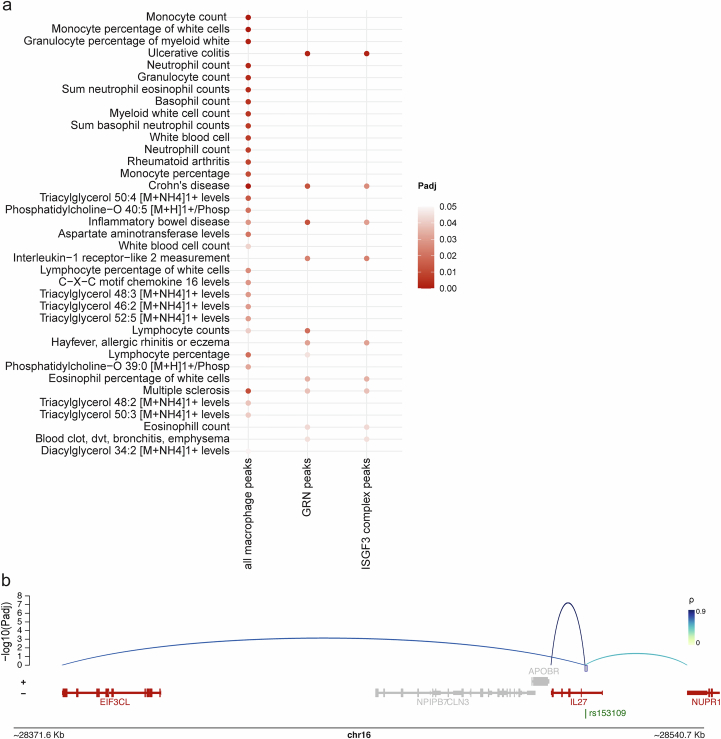
Additional results from linkage disequilibrium score regression (LDSC) analysis, including enrichment for all macrophage peaks and a detailed example of the IL-27 locus. **a**, Identification of diseases and traits associated with genetic variants enriched in open regions of all all macrophage peaks, all macrophage polarization eGRN peaks and a highly connected regulon in the eGRN (the STAT1/STAT2/IRF9 - putative ISGF3 complex) using linkage disequilibrium score regression (LDSC) (FDR corrected p-values). **b**, Macrophage polarization eGRN peak-gene interactions for the peak within IL27 intersecting with SNP rs153109 (FDR corrected p-values for peak-gene interactions).

Overall, accessible chromatin regions in our macrophage dataset were enriched for white blood cell count traits and autoimmune diseases (Extended Data Fig. 8a). After correcting for general cell type effects (Methods), the eGRN peaks were enriched for immune-related diseases including inflammatory bowel disease (IBD) and two of its subtypes (ulcerative colitis (UC) and Crohn’s disease (CD)), hay fever and multiple sclerosis (Fig. 3f). To identify potential regulatory variants, we mapped putative genome-wide significant single-nucleotide polymorphisms (SNPs) (*P* < 10^−6^) for the top heritability-enriched diseases and traits (*P* < 0.1), identifying 112 unique SNPs that directly overlap with the eGRN peaks (Supplementary Table 7). For UC, the most significant overlapping SNP was rs153109, located in an intron of *IL-27* (Extended Data Fig. 8b). IL-27 is mainly produced by antigen-presenting cells including macrophages and has been implicated in the regulation of the immunological response in IBD^45^ and is a potential drug target for IBD^46^. In our eGRN, this *IL-27*-associated peak is regulated by NFKB1. Interestingly, a *NFKB1* knockout in a THP1 monocyte cell line showed downregulation of IL-27, providing further evidence for its regulatory function^47^.

To zoom in further, we tested heritability enrichment for the peaks within the putative ISGF3 regulon. Although this subset consisted of only 1,673 peaks versus 3,217 for the full eGRN, the enrichments for autoimmune diseases remained significant (Fig. 3f), suggesting that ISGF3 plays an important role in macrophage-associated autoimmune diseases. Indeed, disease-associated SNPs mapping specifically to the regulatory regions inferred to be targeted by ISGF3 prioritized 19 genes for IBD, 16 for CD and 37 for eosinophil counts (Methods and Supplementary Table 8). We then calculated the expression activity for these disease-associated genes (Methods) and projected them along the M1 sustained response factor. The IBD and CD gene sets showed a stronger increase in activity over time compared with both the full ISGF3 regulon and ‘eosinophil count’ gene set, suggesting that the polarized M1 state is particularly important for these diseases (Fig. 3g). Another example bridging the M1 polarization and genetic risk is rs4810485, a SNP within a peak putatively targeted by ISGF3. This SNP, located in an intron of *CD40*, is associated with IBD, CD and UC. In our eGRN, this *CD40* intronic peak is linked to *CD40*, *PLTP*, *NEURL2* and *SLC35C2* (Fig. 3h). This SNP rs4810485 is linked to *CD40* as an expression, protein and splice quantitative trait locus in blood and monocyte-specific datasets, as well as to *PLTP* as a blood expression quantitative trait locus^48^ (Supplementary Table 9). CD40, a cell surface receptor expressed by antigen-presenting cells including macrophages, contributes T cell activation and is probably involved in the pathogenesis of IBD^49^. *CD40* expression can be induced by IFN-γ stimulation in macrophages, a process thought to be involved in the initiation of chronic inflammation in IBD^50,51^.

### SUM-seq recapitulates drivers of T cell differentiation

**Fig. 4:**
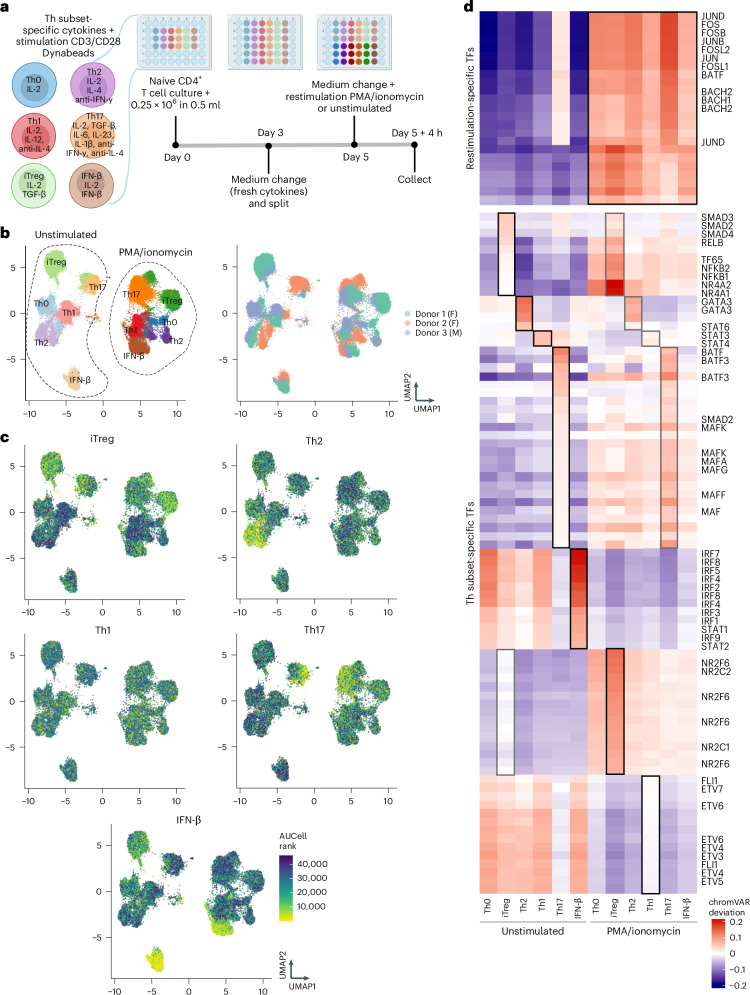
SUM-seq data validate the identity of human T cells differentiated to diverse Th subsets and reveal subset-specific and restimulation-responsive TFs. **a**, A schematic overview of the human primary T cell differentiation experiment to Th subsets. **b**, WNN UMAP projection of integrated SUM-seq data of Th subsets. Cells are annotated and labeled according to their Th subset identity (left) or donor of origin (right). F, female donor; M, male donor. **c**, WNN UMAP projection of integrated SUM-seq data from Th subsets. Cells are colored according to their ranked AUCell scores, which quantify the expression of marker genes specific to each Th subset. **d**, chromVAR deviations (used as proxy for TF motif activity) of identified Th subset-specific and restimulation-specific TFs (bottom and top, respectively). Only significant TFs (Wilcoxon rank-sum test, FDR-corrected *P* value < 0.001) with a positive logFC, at least 10 RNA counts and a high distinctive value (AUC >0.9 for stimulation, AUC >0.7 for subset) are plotted. The black boxes highlight restimulation and subset-specific TFs that passed the abovementioned thresholds and gray boxes highlight their stimulation counterpart when the activity pattern was recapitulated.

To demonstrate the use of SUM-seq in primary samples, we applied it to human CD4^+^ T cells. T cells can take on a number of different roles, depending on the cytokines in their microenvironment^52^. Here, we modeled T cell activation of diverse T helper (Th) subtypes by culturing naive CD4^+^ T cells from healthy donors with anti-CD3/anti-CD28 coated beads in the presence of distinct cytokine cocktails (Methods). Briefly, naive CD4^+^ T cells were isolated from peripheral blood mononuclear cells (PBMCs) obtained from buffy coats from three healthy human donors (two females and one male), and differentiated into Th0, induced regulatory T cells (iTregs), Th2, Th1, Th17 and IFN-β-activated subsets using a previously described protocol^52^. Following 5 days of differentiation, T cells were left unstimulated or restimulated with phorbol 12-myristate 13-acetate (PMA) and ionomycin (Fig. 4a and Methods), and subjected to SUM-seq (36 plex).

Following QC filtering (Methods), we retained 48,153 nuclei containing both RNA and ATAC information. For the snATAC modality, nuclei showed an average of 8,967 unique fragments and a TSS score of 8.9 (with 35,000 reads per cell). For the snRNA-seq, nuclei had an average of 1,197 UMIs and 905 genes (with 20,000 reads per cell). The TSS scores, number of UMIs and number of fragments in peaks were comparable across T cell subsets and donors (Supplementary Fig. 1a). We observed a notable donor-dependent difference in donor 2, where the iTregs (anti-inflammatory) cluster together with the Th17 (proinflammatory) in both stimulated and restimulated conditions (Fig. 4b and Supplementary Fig. 1b). This clustering may indicate a more proinflammatory immune profile in donor 2, with increased functional or phenotypic overlap between Th17 and iTreg subsets. This underscores the role of individual immune profiles in shaping T cell subset differentiation outcomes and highlights the importance of including multiple donors to study T cell differentiation.

Using naive Th cell state-specific gene signatures^52^ (Methods), we observed that all Th subsets, with exception of Th1, expressed distinct markers consistent with those identified previously^52^ (Fig. 4c). To validate the identity of the Th1 subset, we expanded the Th1 gene signature to additional marker genes identified in the same study (Methods), thereby confirming the identity of our Th1 cells (Supplementary Fig. 1c). We also calculated differentially expressed genes per subset from our dataset (Supplementary Table 10). While the expression profiles have been characterized before^52^, the additional chromatin levels from SUM-seq allowed us to identify differentially accessible regions and TFs that drive the differentiation toward each Th subset. Across the unstimulated and restimulated conditions, each Th subset exhibited thousands of uniquely accessible regions (between 7,303 and 119,053; Supplementary Fig. 2a,b and Supplementary Table 11).

We next classified TFs based on the combination of their activity in each Th subset and in response to PMA/ionomycin stimulation into (1) stimulation-responsive activators (Fig. 4d, top) and (2) Th subset-specific activators (Fig. 4d, bottom, and Supplementary Table 12). For example, the AP-1 complex members (FOS, JUN and BATF family) showed increased activation in all Th subsets upon restimulation, in line with their well-established roles in T cell activation^53^. Conversely, we also observed subset-specific TFs: iTreg (TF65 (ref. ^54^), SMAD2/3/4 (ref. ^55^) and NR4A1/2 (refs. ^56,57^)), Th2 (GATA3 (ref. ^58^)), Th1 (STAT4 (ref. ^59^)), Th17 (BATF^60^ and MAF^61^) and IFN-ϐ (STAT1/2 and IRF family^62^). Most TFs displayed Th subset specificity both before and after stimulation (Fig. 4d).

Together, these findings highlight the value of chromatin-level analysis in elucidating the regulatory mechanisms that define and maintain T cell subset identity and function. Moreover, the alignment with known T cell biology highlights the robustness of SUM-seq, positioning it as a reliable method for capturing the regulatory landscape within primary human cells.

### SUM-seq following CRISPR screens maps cell fate programs

**Fig. 5:**
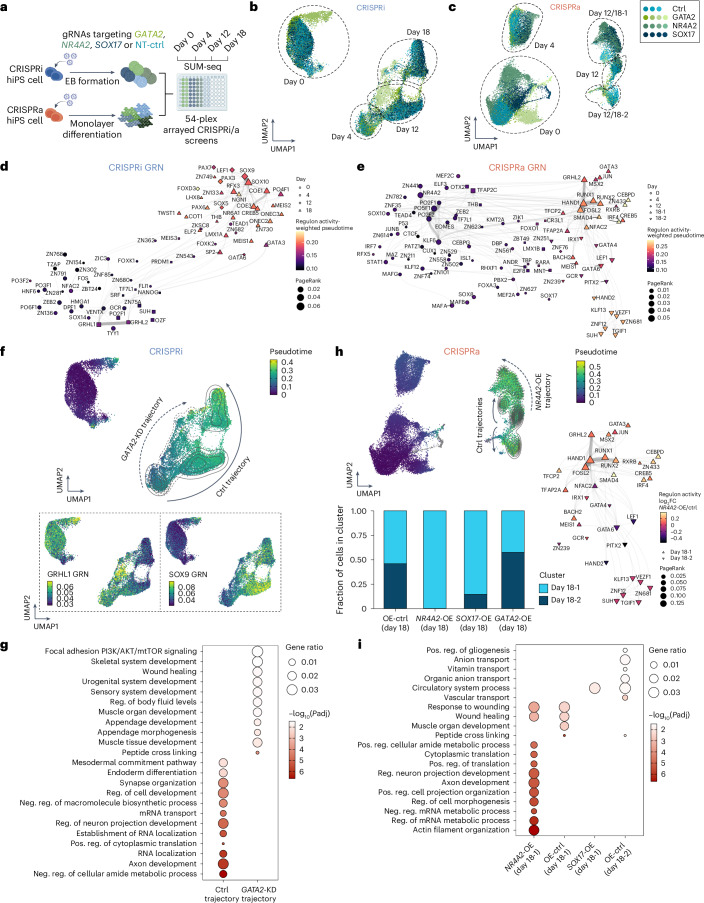
SUM-seq paired with CRISPRi/a characterizes the roles of GATA2, NR4A2 and SOX17 on regulatory network dynamics along spontaneous hiPS cell differentiation. **a**, A schematic overview of the 54-plex arrayed CRISPRi/a screens targeting lineage TFs *GATA2*, *NR4A2* and *SOX17* along a time course of spontaneous differentiation (*n* = 2 clones per CRISPR modality; 1–2 gRNAs per target and nontargeting control (NT-ctrl)). Created with BioRender.com. **b**,**c**, WNN UMAP of the CRISPRi/a SUM-seq screens along spontaneous differentiation of EBs (**b**) or within a monolayer (**c**) colored by the combination of their cluster and perturbation (sample index; collapsed by targeted gene). **d**,**e**, UMAP representation of GRNs inferred for CRISPRi (**d**) and CRISPRa (**e**) cells. GRN TFs are colored by the multiplication of their activity (AUCell) and pseudotime across all cells (approximating the pseudotime point of their highest activity), while shape corresponds to the day of the highest regulon AUCell score. The line thickness indicates the number of shared targets of regulon genes. **f**, Top: UMAP of CRISPRi cells colored by pseudotime and overlaid with contour lines indicating the location of *GATA2*-KD (dashed lines) and control cells (solid lines) from day 4 to 18. Bottom: UMAPs showing higher AUCell scores for the GRHL1 regulon in the *GATA2*-KD trajectory (left) and SOX9 regulon in the control trajectory (right). **g**, Gene Ontology enrichment (hypergeometric test) for differentially expressed genes between the CRIPSRi control and *GATA2*-KD trajectories. Size represents gene ratio and color the −log_10_ FDR-corrected *P* value. **h**, Top: UMAP of CRISPRa cells colored by pseudotime and overlaid with contour lines indicating the location of *NR4A2*-OE (dashed lines) and control (solid lines) cells from day 12 to 18. Bottom left: bar plot depicting the fraction of control, *NR4A2*-OE, *SOX17*-OE and *GATA2*-OE cells in the two day 18 subpopulations. Bottom right: UMAP representation of GRNs inferred for day 18 of the CRISPRa screen. GRN TFs are colored by the log_2_ fold change in regulon AUCell score between the *NR4A2*-OE and control groups. Shape corresponds to the day of highest regulon AUCell score. **i**, Gene Ontology and WikiPathways enrichment (hypergeometric test) for differentially expressed genes for *NR4A2*-OE, *SOX17*-OE and control groups of cells in the day 18-1 subpopulation. Size represents gene ratio and color the −log_10_ FDR-corrected *P* value. Pos., positive; neg., negative; reg., regulation.

**Extended Data Fig. 9:**
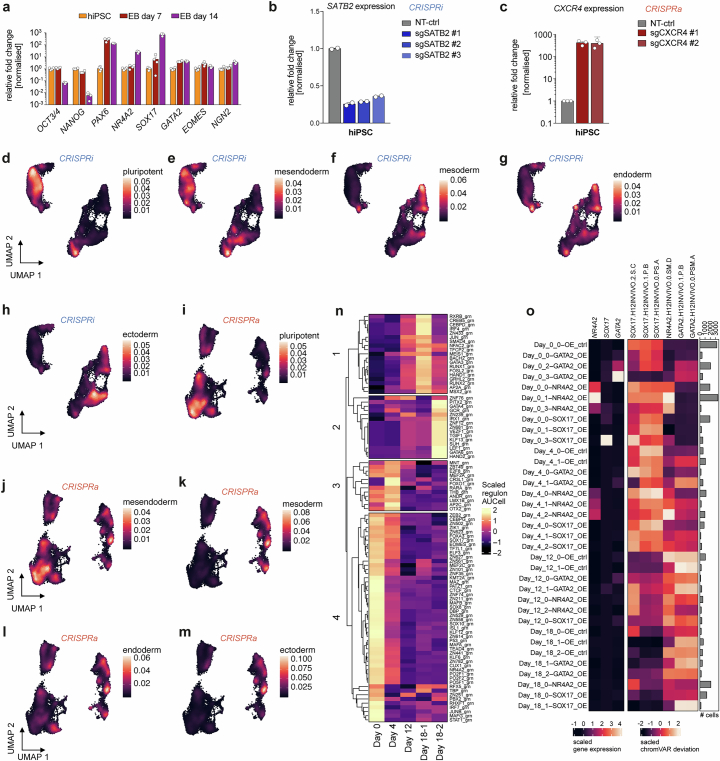
Emergence of distinct lineage states and transcription factor - regulon activities over the course of spontaneous hiPSC differentiation. **a**, Confirmation of target gene expression over a time course of spontaneous hiPSC differentiation as EBs. Relative expression levels were measured via qPCR following the ∆∆Ct method, normalized to *GAPDH* and *ACTB* and experimental control (day 0 hiPSCs). Bar plots show mean ± s.e.m. (*n* = 4 independent replicates). **b, c**, Validation of CRISPRi/a machineries individually targeting *SATB2* (**b**, CRISPRi) or *CXCR4* (**c**, CRISPRa) with 2-3 gRNAs. Relative expression measured as described in a, while normalizing to nontargeting (NT)-ctrl group. **d–m**, UMAP showing the differentiation time course of cells in the CRISPRi EB differentiation (**d**–**h**) and CRISPRa monolayer differentiation (**i**–**m**). Cells are coloured by the weighted kernel density estimation of AUCell scores of pluripotency (**d,i**), mesendodermal (**e,j**), mesodermal (**f,k**), endodermal (**g,l**), and ectodermal (**h,m**) genes (gene list derived from Dobner et al. 2024). **n**, Heatmap showing the scaled regulon AUCcell scores across the differentiation timepoints (x-axis) in the monolayer CRISPRa screen. **o**, Heatmap showing the scaled expression of CRISPRa target genes (left panel), scaled TF motif activity of the CRISPRa targets (center panel), and total nuclei counts per group (right barplot) across differentiation timepoint, and CRISPRa-perturbation assigned groups.

To showcase the multiplexing capacity of SUM-seq, we combined it with arrayed CRISPR screens modulating the expression of key lineage TFs (GATA2–mesoderm^63^, SOX17–endoderm^64^ and NR4A2–neuroectoderm^65^) in hiPS cells via CRISPR interference (CRISPRi) or activation (CRISPRa) over a time course of spontaneous differentiation (days cultured in vitro: 0, 4, 12 and 18; gRNA sequences in Supplementary Table 13). After confirming the expression of the candidate TFs during an unperturbed differentiation time course (Extended Data Fig. 9a) and validating CRISPRi/a functionality (Extended Data Fig. 9b,c), we applied CRISPRi in embryoid body (EB) differentiation and CRISPRa in monolayer differentiation. SUM-seq was performed on hiPS cells and differentiated cells (days 0, 4, 12 and 18), achieving a 54-plex experiment with approximately 150,000 nuclei loaded onto a single 10x Chromium lane (Fig. 5a). We recovered gene expression and chromatin accessibility from 56,652 nuclei with 1,402 UMIs and 982 genes per cell, 4,497 fragments in peaks per cell and an average TSS score of 8.4 (average 17,500 reads per cell). Increased intersample variation was observed, probably due to the cell type heterogeneity of spontaneously differentiating hiPS cells (Supplementary Figs. 3a,b and 4).

Using literature-derived gene signatures for mesodermal, endodermal and ectodermal fates^66^, we observed clear transitions toward lineage-specific fates over time (Fig. 5b,c and Extended Data Fig. 9d–m), with distinct bifurcations at day 18 in monolayer cultures (CRISPRa; Fig. 5c). GRN analysis, visualized using UMAP, revealed subnetworks corresponding to specific differentiation stages (CRISPRi; Fig. 5d and CRISPRa; Fig. 5e). EBs mainly followed neurogenic (NGN1 (ref. ^67^)) and neural crest (SOX9/10 and FOXD3 (ref. ^68^)) trajectories, while monolayer cultures showed a sequential activation of pluripotency (day 0: KLF6 and OCT4), trophectoderm (day 4: TFAP2C^69^), and mesodermal and neural crest (days 12 and 18: HAND1/2 (ref. ^70^)) TFs (Supplementary Tables 14 and 15). By day 18, two monolayer sub-GRNs emerged (Fig. 5e): day 18-1 cells showed a mixed activity for lateral mesodermal–hemogenic and neural crest fate regulons such as of HAND1, MSX2 and RUNX1/2, while day 18-2 cells showed increased activity for cardiac fate regulons of HAND2, KLF13 and VEZF1, capturing the bifurcation of early mesodermal lineages and general mesenchymal fate acquisition (Fig. 5e and Extended Data Fig. 9n).

**Extended Data Fig. 10:**
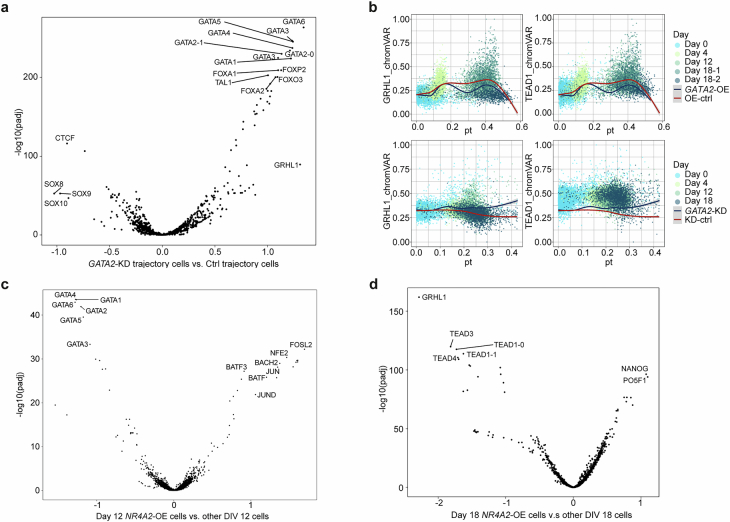
Differential TF and regulon activity captured by the arrayed CRISPRi/a screens exemplified by *GATA2* repression and *NR4A2* overexpression. **a**, Volcano plot showing differential TF motif activity (chromVAR z-scores) between cells in the *GATA2*-KD trajectory and control cells. x-axis is log2(fold change) values and y-axis -log10(FDR corrected p-values) (Wilcoxon rank-sum test). Most significant and most differentially active TFs are highlighted. **b**, GRHL1 (left panels) and TEAD1 (right panels) TF motif activity (chromVAR score, y-axes) along pseudotime (x-axes; pt: pseudotime score) in the CRISPRi EB (top panels) and CRISRPa monolayer (bottom panels) screens. Generalized linear model fitted lines represent the trend in the *GATA2*-KD/OE (blue) and control (red) groups. **c,d**, Volcano plot showing differential TF motif activity (chromVAR z-scores) between cells in Day 12 *NR4A2-overexpressing* (**c**) control and Day 18 (**d**) cells. x-axis is log2(fold change) values and y-axis -log10(FDR corrected p-values) (Wilcoxon rank-sum test). Most significant and most differentially active TFs are highlighted.

CRISPR perturbations impacted both the expression and activity of the targeted TFs (Extended Data Fig. 9o), revealing distinct cell states over time (Fig. 5b,c and Supplementary Tables 16–19). In line with the role of GATA2 in promoting the hemato-endothelial lineages and repressing, for example, the cardiac fate^63,71^, *GATA2*-knockdown (KD) decreased expression of lateral mesodermal genes, while increasing expression of non-hemato-endothelial lineages, including skeletal and muscle development-associated genes (Fig. 5f,g). In contrast, control cells showed higher expression of neuronal genes associated with axon development and synapse organization. At the regulatory level, *GATA2*-KD cells exhibited increased TF motif activity of GRHL1, TEAD1, GATA and FOX family members, while SOX9, SOX10 and CTCF motif activity decreased (Extended Data Fig. 10a). GRN analysis further confirmed increased GRHL1 regulon activity in *GATA2*-KD cells and increased SOX9 activity in control cells (Fig. 5d). In line, *GATA2* overexpression (OE) showed the opposite effect, decreasing GRHL1 and TEAD1 motif accessibility along the developmental trajectory (Extended Data Fig. 10b).

Similarly, *NR4A2*-OE skewed cells toward ectoderm-like fates (18-1) at day 18 (Fig. 5e,h), underscoring its role in promoting neuroectodermal fate^72^. These cells showed upregulation of neuronal genes (Fig. 5i) and increased motif and regulon activity of proneurogenic TFs such as JUN and FOS at days 12 and 18, with concomitant reduction of mesoderm and endoderm-associated GATA TFs^73^ (Fig. 5h and Extended Data Fig. 10c). Notably, *NR4A2*-OE at day 18 increased motif activity of pluripotency TFs such as OCT4 and NANOG (Extended Data Fig. 10d), which may recapitulate the reactivation of pluripotency programs at the initiation of neural crest differentiation^74^.

## Discussion

We developed SUM-seq, a single-cell profiling method that captures chromatin accessibility and gene expression across a highly multiplexed set of samples, outperforming previously published scalable high-throughput methods^6,8–13,75^. SUM-seq generates high-quality data from fresh and cryopreserved samples, making it adaptable for studies that require multicenter or asynchronous sampling. While the current format measures chromatin accessibility and gene expression, the strategy can be adapted for the measurement of other omic layers such as surface proteomic readouts^76^, TF binding and histone modifications (Cut&Tag^77^), DNA methylation (sci-MET^78^) and whole-genome sequencing (s3-WGS^79^).

We demonstrated the use of SUM-seq for time-course experiments (macrophage polarization), cytokine screens in primary cells (PBMC-derived T cells) and arrayed CRISPRi/a screens (induced pluripotent stem cells). This allowed defining core GRNs that drive cell type-specific functions and developmental processes. When integrated with genetic disease evidence, SUM-seq provides a framework for linking noncoding genetic variants to their target genes and molecular pathways. Such insights offer intriguing opportunities to enhance our understanding in fields such as developmental biology and especially genetics of complex human diseases.

While we have shown that SUM-seq can be applied in multiple experimental setups, there are also limitations. Some cell types are prone to clumping during the protocol, which prevented us from including PBMCs in our application. Performing SUM-seq on sorted PBMC cell fractions revealed that primarily monocytes undergo clumping, whereas T and B cells exhibited greater robustness (data not shown). Thus, specific cell types may be more susceptible to the handling demands inherent to this method. Second, SUM-seq does not reach the gene expression complexity of lower throughput droplet-based single-cell multiomic methods (10x Multiome or ISSAAC-seq). However, the chromatin accessibility complexity does match existing platforms. We argue that the substantial improvement in cost-efficient scalability and throughput will often outweigh the lower gene expression complexity as also argued in ref. ^80^, but this may depend on the research question at hand.

In conclusion, our study showcases the effective implementation of SUM-seq and suggests applicability to diverse experimental settings that demand gene regulatory analyses at single-cell resolution. Specifically, SUM-seq is a promising tool for drug or perturbation screens and large-scale atlas projects, underscoring its potential as a versatile and powerful technique across a spectrum of biological studies. Overall, we envision SUM-seq as an easily adoptable, scalable method for projects requiring multiomic single-cell profiling up to millions of cells from hundreds of samples.

## Methods

### Species-mixing benchmarking experiments

Human myelogenous leukemia cells (K562, DSMZ no. ACC 10) were cultured in RPMI 1640 medium (Gibco, 11875093) supplemented with 10% FBS (Gibco, 10270-106), 100 U ml^−1^ penicillin/streptomycin (PenStrep, Gibco, 15140122) and 1× non-essential amino acids (NEAAs, Gibco, 11140050) at 37 °C with 5% CO_2_. Mouse fibroblast cells (NIH-3T3, DSMZ no. ACC 59) were cultured in DMEM medium (high glucose, Gibco, 11965092) supplemented with 10% FBS, 100 U ml^−1^ PenStrep and 1× NEAAs at 37 °C with 5% CO_2_. The multiplexing capacity of SUM-seq was leveraged to test parameters and optimize the protocol, including the impact of cryopreservation, inclusion of PEG to the RT step and measures to mitigate Tn5 hopping, including the utilization of a blocking oligonucleotide and reduction of thermal amplification cycles during microfluidic barcoding. For each condition, two separate sample indices were used.

### Macrophage differentiation

hiPS cells (CESCG-295; male, PBMC derived; provider: Dr. Michael Snyder, Stanford University) were cultured in Essential 8 medium (Gibco, A1517001) with vitronectin (Thermo Fisher, A14700) coating. hiPS cells were differentiated to macrophages as previously described^82^. In brief, 4 million hiPS cells were resuspended in EB medium (Essential 8 medium, 50 ng ml^−1^ Recombinant Human BMP4 (Peprotech, 120-05ET), 50 ng ml^−1^ Recombinant Human VEGF (Peprotech, 100-20), 50 ng ml^−1^ Recombinant Human SCF (Peprotech, 300-07)) with 10 μM Y-27632 (AbCam Biochemicals, ab120129), seeded in 400-microwell Aggrewells (Stemcell Technologies, 34450) and centrifuged at 100*g* for 3 min to distribute to microwells. Seventy-five percent of the medium was replaced on the next two consecutive days. On the third day, EBs were transferred to a low-attachment plate (Sigma-Aldrich, CLS3471-24EA) and cultured for two additional days. On day 5, EBs were transferred to culture flasks and cultured for 1 week in factory media (X-VIVO-15 (Lonza, BE02-060F) 1% GlutaMax (Thermo Scientific, 35050061), 1% PenStrep, 50 µM β-mercaptoethanol, 50 µg ml^−1^ Normocin (Invivogen, ant-nr-05), 100 ng ml^−1^ Recombinant Human M-CSF (Peprotech, 300-25) and 25 ng ml^−1^ Recombinant Human IL-3 (Peprotech, 200-03)). Fresh media were added weekly. After approximately 4 weeks, formed macrophage precursors were collected weekly, taking approximately 1/4 of the total culture volume. For terminal differentiation to macrophages, precursors were resuspended in macrophage media (X-VIVO-15, 1% GlutaMax, 1% PenStrep and Recombinant Human M-CSF) and plated at a density of 160.000 cells per cm^2^ for 7 days.

### Polarization to M1 or M2 macrophages

On day 7 macrophages were polarized to M1 or M2. For M1 polarization, macrophage media supplemented with 20 ng ml^−1^ human recombinant IFN-γ (PeproTech, 300-02) and 25 ng ml^−1^ LPS (Invivogen, tlrl-3pelps). For M2 polarization, macrophage media supplemented with 20 ng ml^−1^ recombinant human IL-4 (PeproTech, 200-04). Cells were collected after 24, 10, 6 and 1 h, including an undifferentiated control (M0) in which medium was replaced by fresh macrophage medium.

### CD4^+^ T cell isolation and culture

Buffy coats from healthy human donors were obtained from the DRK-Blutspendedienst Baden-Württemberg – Hessen. PBMCs were isolated by density-gradient centrifugation using the Ficoll-Paque Plus solution (GE Healthcare Life Sciences, GE17-1440-02), and stored in liquid nitrogen until T cell isolation. Naive CD4^+^ T cells were isolated using the EasySep Human Naive CD4^+^ T cell Isolation kit II (Stemcell Technologies, 17555) with ‘The Big Easy’ EasySep Magnet (Stemcell Technologies, 18001). Naive T cells were cultured in ImmunoCult-XF T cell Expansion medium (Stemcell Technologies, 10981) in the presence of Human T-Activator CD3/CD28 Dynabeads (Gibco, 11131D), supplemented with Th subset-specific cytokine mixes (below), and 1% PenStrep.

- Th0: 10 ng ml^−1^ IL-2 (Peprotech, 200-02).
- Th2: 10 ng ml^−1^ IL-2, 10 ng ml^−1^ IL-4 (Preprotech, 200-04), 1 μg ml^−1^ anti-IFN-γ (R&D systems, MAB285-SP).
- Th1: 10 ng ml^−1^ IL-2, 50 ng ml^−1^ IL-12 (Miltenyi Biotec, 130-096-705), 1 μg ml^−1^ anti-IL-4 (R&D systems, MAB204-100).
- Th17: 10 ng ml^−1^ IL-2, 5 ng ml^−1^ TGF-β1 (Peprotech, 100-21), 50 ng ml^−1^ IL-6 (Preprotech, 200-06), 20 ng ml^−1^ IL-23 (Preprotech, 200-23), 10 ng ml^−1^ IL-1β (Preprotech, 200-01B), 1 μg ml^−1^ anti-IFN-γ, 1 μg ml^−1^ anti-IL-4.
- iTreg: 10 ng ml^−1^ IL-2, 5 ng ml^−1^ TGF-β1.
- IFN-β: 10 ng ml^−1^ IL-2, 10 ng ml^−1^ IFN-β (Preprotech, 300-02BC).

After 3 days of culture, the medium was changed to fresh medium supplemented with subset specific cytokines, and for every condition, cells were split into two new wells. After 5 days, the medium was again changed and the cells were left unstimulated or stimulated with 50 ng ml^−1^ PMA (Sigma-Aldrich, P8139-1MG) and 1 µg ml^−1^ ionomycin (Sigma-Aldrich, I0634-1MG) for 4 h.

### Arrayed CRISPRi/a screen

Clonal cell lines were established integrating dCas9 fusion proteins (KRAB–MeCP2 for CRISPRi, VP64 for CRISPRa) into the *CLYBL* safe-harbor locus using TALE nucleases in NGN2-hiPS cells. Guide RNAs targeting the TSS of *NR4A2*, *SOX17* and *GATA2* (gRNA sequences from Sanson et al.^83^ and listed in Supplementary Table 13) were cloned into a modified CROP-seq (CROP-seq-tagBFP-puroR for CRISPRi) or the pXPR_502 lentivectors (Addgene 96923, CRISPRa) via BsmBI (NEB, R0739) restriction digest. Lentiviral particles were packaged using calcium-phosphate transfection (Takara, 631312) in HEK293T cells and a second-generation lentiviral system (pMD2.G, Addgene 12259; psPAX2, Addgene 12260). Lentiviral particles were quantified performing serial dilution assays in combination with puromycin resistance using the cell titer blue assay (Promega, G8080).

Clonal CRISPRi/a hiPS cell lines (*n* = 2 clones each) were transduced with nontargeting control or targeting gRNAs at an multiplicity of infection of 0.4 and selected with puromycin (1 µg ml^−1^ after 24 h, increasing up to 2.5 µg ml^−1^ for additional 3 days). After recovery, hiPS cell clones were pooled and either collected and cryopreserved in CryoStor CS10 (Stemcell Technologies, 07959; day 0 hiPS cell samples) or plated (2.5 × 10^5^ cells per condition) to induce spontaneous differentiation in KSR medium (CRISPRi-SUM-seq as EBs and CRISPRa-SUM-seq as monolayers; KSR medium: knockout DMEM/F12 (Gibco, 12660012), 2 mM GlutaMAX, 1× NEAAs, 20% knockout serum replacement (Gibco, 10828010) and 0.1 mM β-mercaptoethanol (Merck-Millipore)). Vitronectin-coated tissue-culture (TC) plates were used for the CRISPRa monolayer differentiation, while non-TC plates and dishes were used for the CRISPRi EB differentiation. At the day of plating, KSR medium was supplemented with Y-27632 (10 µM). The next day, medium was replaced with fresh KSR medium. Medium changes were performed every other day. EBs were transferred to 15 ml conical tubes and allowed to sediment at room temperature for 5 min. The supernatant was replaced with fresh KSR medium and cells were transferred onto non-TC treated plates and maintained at 37 °C, 5% CO_2_. On day 12 of differentiation, the KSR medium was replaced with Essential 6 medium (Gibco, A1516401). EBs and cell monolayers were collected at 4, 12 and 18 days following spontaneous differentiation and cryopreserved in CryoStor CS10 until the day of SUM-seq library preparation.

### Nuclei preparation

Mouse NIH-3T3 cells were washed once with PBS and dissociated with Accutase (Stem Cell Technologies, 07920). Two million NIH-3T3 and K562 cells were collected and fixed in a 3% glyoxal solution (40% glyoxal (Merck, 128465) and 0.75% acetic acid; adjusted to pH 5 by addition of 1 M NaOH) for 7 min at room temperature. For cryopreservation, cells were washed after fixation with RSB–1% BSA-RI (10 mM Tris–HCl pH 7.5, 10 mM NaCl, 3 mM MgCl_2_, 1 mM dithiothreitol (DTT), 1% BSA, 20 µg ml^−1^ in-house produced RNasin (referred to RNasin hereafter)) and slowly frozen (freezing buffer: 50 mM Tris–HCl pH 7.5, 5 mM MgAc, 0.1 mM EDTA and 25% glycerol). On the experiment day, fresh cells were processed as described above, while cryopreserved cells were thawed slowly on ice and washed with RSB–1% BSA-RI. Nuclei were extracted with 1× lysis buffer (10 mM Tris–HCl pH 7.5, 10 mM NaCl, 3 mM MgCl_2_, 0.1 % Tween20, 20 µg ml^−1^ RNasin, 1 mM DTT, 1% BSA, 0.025% IGEPAL CA-630 and 0.01% Digitonin) incubating samples for 5 min on ice. Next, nuclei were washed with wash buffer (10 mM Tris–HCl pH 7.5, 10 mM NaCl, 3 mM MgCl_2_, 0.1% Tween20, 20 µg ml^−1^ RNasin, 1 mM DTT and 1% BSA) and filtered through a 40 µm cell strainer (Falcon, 352340).

Macrophages were collected by replacing the medium with ice-cold PBS–1% BSA and 2 mM EDTA and pipetting. Nuclei were extracted by resuspending the cell pellet in a 1× lysis buffer for 4 min on ice and washed with a wash buffer. Nuclei were fixed with a 3% glyoxal solution for 7 min at room temperature and washed with RSB–1% BSA-RI.

T cells were collected by first removing CD3/CD28 Dynabeads on a magnet, whereafter cells were fixed in 3% glyoxal pH 5.0 solution for 7 min at room temperature. Nuclei were extracted by resuspending cells in 1× lysis buffer, incubated for 1 min on ice, and washed with 1× wash buffer. Nuclei from a subset of samples were quantified using 4,6-diamidino-2-phenylindole (DAPI) staining, and downstream library preparation was performed with approximately 40,000 nuclei per sample.

Cryopreserved cells of the arrayed CRISPRi/a screen were thawed in a water bath at 37 °C and transferred to 1.5 ml tubes with PBS–1% BSA. EBs were allowed to sediment by gravity on ice, while monolayer samples were centrifuged (300*g*, 5 min, 4 °C). Cells were washed with PBS–1% BSA and nuclei isolated with 1× lysis buffer. EBs were incubated on ice for 1 min and dounced with a 7 ml douncer until visible cell aggregates were resolved, transferred back into the respective 1.5 ml tube and incubated on ice for an additional 3 min. Monolayer samples were resuspended in 1× lysis buffer and incubated on ice for 5 min. Nuclei were washed with 1× wash buffer and centrifuged (500*g*, 5 min, 4 °C), strained through a 40 µm mesh filter and fixed in 3% glyoxal solution (7 min incubation at room temperature). Following two wash steps in 1×RSB–1% BSA-RI, nuclei were counted for each sample with DAPI and library preparation was performed with 40,000 nuclei per sample.

### Transposome generation

Tn5 was produced in-house according to a previously described protocol^84^. For transposome assembly, 100 µM of oligonucleotides with the Nextera Read1 sequence and 100 µM of oligonucleotides with a Read2-sample_index-spacer structure were annealed with 100 µM mosaic end-complement oligonucleotides with a 3′ dideoxynucleotide end (ddc) at a 1:1:2 ratio by heating for 95 °C for 3 min followed by cooling to 25 °C at a ramp rate of −1 °C min^−1^. Annealed oligos were mixed with an equal volume of 100% glycerol and stored at −20 °C until use. Finally, the annealed oligos were mixed with the in-house produced Tn5 (at 1 mg ml^−1^ in 50 mM Tris, 100 mM NaCl, 0.1 mM EDTA, 1 mM DTT, 0.1% NP-40 and 50% glycerol), at a 1:1 ratio and incubated for 30–60 min at room temperature. For the species mixing, T cell stimulation and arrayed CRISPR screen experiments, annealed oligos were first diluted with H_2_O at a 1:1 ratio before generating the transposomes. Assembled Tn5 was stored at −20 °C until use.

### SUM-seq procedure

#### DNA transposition

Isolated and fixed nuclei were resuspended in a transposition mix containing 38.8 mM Tris-acetate, 77.6 mM potassium acetate, 11.8 mM magnesium acetate and 18.8% dimethylformamide supplemented with 0.005× protease inhibitor cocktail (Roche, 11697498001), 0.4 U µl^−1^ SUPERaseIn (Thermo Fisher, AM2694), 1.2 U µl^−1^ RiboLock (Thermo Fisher, EO0382) and the barcoded Tn5 transposomes (1:10 (v/v); 938 µM) and incubated at 30 °C for 30 min with shaking at 400 rpm. The reaction was terminated by adding an equal volume of 2× stop buffer (10 mM Tris–HCl pH 7.5, 20 mM EDTA pH 8.0 and 2% BSA).

#### In situ RT

Transposed nuclei were washed three times with RSB–1% BSA-RI and resuspended in RT mix containing 1× RT buffer (50 mM Tris–HCl pH 8.0, 75 mM NaCl, 3 mM MgCl_2_ and 10 mM DTT), 0.5 mM dNTPs, 1 U µl^−1^ Protector RNase inhibitor (Roche, 3335402001), 0.2 U µl^−1^ SUPERaseIn (Thermo Fisher, AM2694), 10 U µl^−1^ Maxima H Minus RT (Thermo Fisher, EP0752) and 5 µM of the respective barcoded oligo-dT primer (*n* = 2 per condition). In the NIH-3T3/K562 species mix, T cell and arrayed CRISPR screening experiments, 12% PEG 8000 (w/v; Jena Bioscience, CSS-256) was included in the RT mix as a crowding agent. For the macrophage experiment, crowding agent PEG was omitted from the RT reaction as we observed formation of white precipitates, which hampered downstream steps. This issue was later resolved by omitting BSA from pre- and post-TR washes. Samples were incubated at 50 °C for 10 min, followed by three cycles (8 °C for 12 s, 15 °C for 45 s, 20 °C for 45 s, 30 °C for 30 s, 42 °C for 2 min and 50 °C for 3 min), and finally 50 °C for 5 min. Following the RT reaction, all samples were pooled in RSB-RI and washed twice with RSB–1% BSA-RI.

#### cDNA/mRNA hybrid tagmentation

Nextera Read1 oligonucleotides (100 µM) were annealed with 100 µM of the Tn5-mosaic end oligonucleotides 1:1 (v/v). Annealed oligos were mixed with the in-house produced Tn5 (1:1, v/v) for 30 min at room temperature and stored at −20 °C until use.

The collected nuclei pool was counted, and the reaction volume was adjusted according to the total number of nuclei (100 µl reaction for each 100,000 nuclei). Nuclei were resuspended in the transposition mix containing 1× transposition buffer and a 1:100 ratio (v/v) of assembled Tn5 (93.8 µM). Tagmentation was performed at 37 °C for 30 min with 400 rpm shaking. The reaction was terminated by adding equal volume of 2× stop buffer. Next, the sample pool was washed twice with RSB–1% BSA.

#### Gap fill and ExoI

The sample pool was resuspended in a mix containing 1× RT buffer (50 mM Tris–HCl pH 8.0, 75 mM NaCl, 3 mM MgCl_2_ and 10 mM DTT), 0.5 mM dNTPs, 8 U µl^−1^ Maxima H Minus RT (Thermo Fisher, EP0752) and 2 U µl^−1^ Thermolabile Exonuclease I (New England Biolabs, M0568S) and incubated for 15 min at 37 °C on a shaking thermoblock at 400 rpm. Afterward the sample was washed twice with RSB–1% BSA filtered through a 40 µm cell strainer and counted.

#### GEM generation

Samples with a high debris were cleaned using a glycerol-based buffer (50 mM Tris–HCl pH 7.5, 5 mM Mg-acetate, 0.1 mM EDTA and 25% glycerol). Nuclei were resuspended in the glycerol-based buffer, layered on top of an additional 500 µl buffer and centrifuged at 800*g* for 15 min. The supernatant was discarded, and the pellet washed twice with RSB–1% BSA and filtered through a 40 µM Flowmi cell strainer (Sigma-Aldrich, BAH136800040). Nuclei were counted and loaded into the Chromium Controller (10x Genomics) according to the 10x Genomics single-cell ATAC standard protocol in a mixture containing barcoding reagent B, reducing reagent B and barcoding enzyme. Apart from the macrophage experiment, this reaction mix was supplemented with ~500 nM of a blocking oligonucleotide, preventing Tn5 barcode hopping in multinucleated droplets. Barcode hopping may occur when residual barcoded Tn5 molecules are carried over to droplets. During droplet barcoding, residual barcoded oligonucleotides are released, which through complementarity to ATAC fragments, can lead to barcode hopping within multinucleated droplets. The blocking oligonucleotide competes with the carry-over barcoded oligonucleotides for binding of ATAC fragments. Their 3′-inverted dT modification prevents further amplification of these fragments. For Chip loading, the standard 10x workflow was followed. In brief, 70 µl of the sample, 50 µl of barcoding gel beads and 40 µl partitioning oil were loaded into the respective channels. The droplet emulsion was collected in PCR tubes (Eppendorf, EP0030124359) and cell barcoding was performed as follows: 5 min at 72 °C, 30 s at 98 °C, 4–12 cycles (98 °C 10 s, 59 °C 30 s and 72 °C 1 min). As a second barcode-hopping mitigation strategy, the number of linear amplification cycles was reduced, decreasing the probability of carry-over barcoded oligonucleotides from binding fragments and erroneously barcoding and exponentially amplifying. Then, 125 µl recovery reagent was added to sample and inverted 10–15 times. After brief centrifugation, 125 µl of the pink oil phase was discarded and the aqueous phase was mixed with a cleanup master mix (182 µl cleanup buffer, 13 µl Dynabeads MyOne Silane and 5 µl reducing agent B (10x Genomics)) and incubated for 10 min at room temperature. Samples were placed on a magnetic separator and washed two times with 80% ethanol before eluting in 42 µl of elution buffer. Finally, samples were subjected to a 1.4× solid-phase reversible immobilization (SPRI) cleanup (Beckman Coulter, B23318).

#### Pre- and final library amplification

Samples were resuspended in pre-amplification master mix containing 1× NEBNext HF 2× PCR Master Mix (NEB, M0541L) and three primers: partial-P5-PTO primer (common for both modalities), TruseqR2 (specific for RNA modality) and ATAC-spacer primer (specific for ATAC modality) each at a final concentration of 500 nM. Samples were incubated as follows: 98 °C 1 min, 5 cycles of 98 °C for 20 s, 63 °C for 20 s and 72 °C for 20 s, and 72 °C for 1 min and then subjected to a 1.2× SPRI cleanup eluting in 42 µl EB buffer (Qiagen). Each sample was split into two fractions and further amplified with modality-specific primers: partial-P5-PTO/P7-index-spacer primer pair for the ATAC libraries and partial-P5-PTO/P7-index-TruseqR2 primer pair for RNA libraries. Both library types were incubated at 98 °C for 1 min, 5 cycles of 98 °C for 20 s, 63 °C for 20 s and 72 °C for 20 s followed by a final extension step at 72 °C for 1 min. To estimate the number of endpoint PCR cycles required for each library, 1/20 of the total library volume was used to perform a qPCR reaction using library-specific primers, and the number of cycles required to reach 50% saturation was calculated. ATAC libraries were subjected to a 1.2× SPRI cleanup and RNA libraries to 0.72× SPRI cleanup. Library concentrations were measured using Qubit 1× dsDNA-HS (Thermo Scientific, Q33230) and fragment distributions were assessed using the Bioanalyzer High Sensitivity DNA kit (Agilent, 5067-4626).

#### Sequencing

Benchmarking and macrophage polarization experiment libraries were sequenced on NovaSeq6000 (Illumina) or NextSeq2000 (Illumina) (ATAC: read 1, 55 cycles; index 1, 11 cycles; index 2, 16 cycles; read 2, 55 cycles; RNA: read 1, 95 cycles; index 1, 6 cycles; index 2, 16 cycles; read 2, 21 cycles). T cell stimulation experiment and the arrayed CRISPRi/a screen libraries were sequenced on the Aviti platform (Element Biosystems) (ATAC: read 1, 79 cycles; index 1, 11 cycles; index 2, 16 cycles; read 2, 79 cycles; RNA: read 1, 95 cycles; index 1, 6 cycles; index 2, 16 cycles; read 2, 21 cycles).

### Data analysis procedures

Data analyses were performed in R v4.2.2 and Python v3.9.13. The pipeline used to preprocess the data is accessible from ref. ^14^, it includes bcl-convert (v4.0.3), chromap (v0.2.3), ArchR (v1.02), bcl2fastq (v2.20.0), Je (v2.0.RC), STARsolo (v2.7.11a) and EmptyDrops (v1.16). All code used to perform data analysis are available from ref. ^14^. Tools and databases used are MACS2 (v2.2.9.1), AUCell (v1.20.2), HOCOMOCO (v12), MOFA (v1.6.0), cisTopic (v0.3), Reactome (v59), monaLisa (v1.8), GRaNIE (v1.5.3), LDSC (v1.01) and dbSNP (v155).

#### ATAC-seq data preprocessing

Base calls were converted to fastq format and demultiplexed by i7, allowing for one mismatch, using bcl-convert (v4.0.3). Demultiplexed reads were aligned to the hg38 genome and fragment file generation was performed with chromap (v0.2.3)^85^. For the species mixture experiment, a combined hg38/mm10 reference was used and fragments aligning to either genome were counted. Cells with >90% of fragments aligning to a single species were considered singlets. Using the R package ArchR (v1.02)^86^, generated fragment counts for each cell were computed in 500 base pair genome bins. Cell barcodes were filtered based on the following thresholds: (1) species mixing dataset, number of fragments per cell (≥1,000) and TSS enrichment score (≥5); (2) macrophages dataset, number of fragments per cell (>3,000), TSS enrichment score (≥3), promoter ratio (≥0.08) and maximum 5 nuclei per droplet; (3) T cell dataset, number of fragments per cell (≥1,000) TSS enrichment score (≥5), fraction of reads in peaks (≥0.35) and promoter ratio (≥0.07) and (4) arrayed CRISPR screen dataset, number of fragments per cell (≥1,000), TSS enrichment score (≥5), promoter ratio (≥0.07) and fraction of reads in peaks (≥0.2).

Peak calling was performed using MACS2. In brief, MACS2 peak caller is used to identify peaks for groups of cells, whereafter an iterative overlap peak merging procedure is done to derive a consensus peakset between all samples. BigWig files for trace plots were generated with ArchR::plotBrowserTrack normalizing for reads in TSS.

#### RNA-seq data preprocessing

Base calls were converted to fastq format using bcl2fastq (v2.20.0) or bcl-convert (v4.0.3). The cell barcode (i5) was concatenated to the sample index and UMI (Read2), whereafter reads were demultiplexed by sample index using Je (v2.0.RC), allowing two mismatches. Read alignment to Hg38 and expression matrix generation was conducted with STARsolo. For the species mixing experiment, a combined Hg38/mm10 reference was used and number of UMIs aligning to each genome was determined for each cell to estimate collision rates. Cells with >80% of UMIs aligning to a single species were considered singlets. For each sample, cell calling was either performed with EmptyDrops (v1.16) or by determining inflection points on a rank versus UMI plot. Additionally, cells were filtered by mitochondrial and ribosomal read percentage. Feature by cell matrices were merged between samples, and features in a minimum of 10–25 cells were retained. Additional filtering was applied to the macrophage data (>150 genes per nuclei and <6 nuclei per droplet), the T cell data (>400 genes per nuclei, >600 and <5,000 unique fragments and <10% mitochondrial reads), and the arrayed CRISPR screen (>200 unique fragments)

#### Comparison with other technologies (K562 and 3T3)

We compared the performance of SUM-seq with dsciATAC-seq^8^ (GSM3507387), SHARE-seq^11^ (GSM4156590, GSM4156594 and GSM4156596 (ATAC), GSM4156602, GSM4156603 and GSM4156605), Paired-seq^12^ (ATAC: GSM3737488, RNA: GSM3737489), scifi-RNA-seq^6^ (GSM5151362), ISSAAC-seq^10^ and MultiPerturb-seq^13^ (GSM8528725) using cell line data. We used K562 and NIH-3T3 data for SUM-seq, sequenced to approximately 30,000 reads per cell for each modality (~66% saturation). For other methods, we used count matrices provided in the indicated repositories. Cell calling for the RNA modality was performed based on UMI rank knee plots or with EmptyDrops, and for the ATAC modality based on the distribution of fragments in peaks. In the case of mixed-species datasets without annotation available, cells with >80% UMIs or >90% fragments in peaks aligning to a single species were considered.

#### Dimensionality reduction and modality co-embedding

The peak matrices were normalized using term frequency-inverse document frequency normalization, followed by singular value decomposition. The number of components considered was based on the proportion of variance explained (range 30–50), discarding the first component due to high correlation with number of fragments per cell.

For the macrophage and EB experiment, the gene expression matrix was normalized by proportional fitting (cell depth normalization to the mean cell depth), followed by logarithmic transformation (log(*x* + 0.5)), and another round of proportional fitting^87^. For the T cell experiment, the gene expression matrix was normalized by logarithmic transformation. Following normalization, principal component analysis was performed. The number of components considered was based on the proportion of variance explained (range 30–50).

Derived low-dimensional representations for each modality was used as input to learn cell modality weights, from which a weighted nearest neighbor (WNN) graph was constructed^88^. The WNN graph was used to define a common UMAP visualization.

#### M1 and M2 signature score generation (macrophages)

We obtained publicly available datasets (GSE159112 (ref. ^89^) and GSE55536 (ref. ^90^)) and used DESeq2 (ref. ^91^) to identify genes that were differentially expressed in M1 and M2 macrophages compared with M0 (adjusted *P* value <0.05 and fold change >0). Genes identified as differentially expressed in both datasets were used as gene sets for input to the ranking based scoring-approach AUCell^92^.

#### MOFA (macrophages)

MOFA^23^ provides a set of latent factors each representing a dimension of variability in the data. For each factor, every cell is assigned a weight, which reflects the cell’s contribution to the inferred axis of variation captured by that factor. Likewise, the positive or negative association of a feature (genes/cistopics) with a given factor are reflected by the value of their signed feature weights for that factor. First, ATAC data were collapsed to *cis*-regulatory topics with cisTopic (v0.3)^93^, determining a suitable number of topics based on the maximum on the second-derivative of likelihood curve and minimum on the model perplexity curve. All topics were used as input for MOFA. For RNA, the top 4,000 most variable genes were used as input.

To determine polarization-associated latent factors, latent factors were correlated with biological metadata (state (M0/M1/M2) and time point) as well as technical metadata (UMI per cell and fragments per cell). Factors with a high correlation against biological metadata, but not technical metadata were chosen as factors for downstream analysis.

#### TF motif accessibility (macrophages, T cells, arrayed CRISPR screen)

Position weight matrices (PWMs) of TF-binding motifs were obtained from HOCOMOCO v12 (in vivo subcollection)^94^. Motif positions in the accessible chromatin regions were determined using the function ArchR::addMotifAnnotations. Deviations and the *z* score of bias-corrected per-cell motif accessibility was calculated with the function ArchR::addDeviationsMatrix, which leverages the chromVAR framework^28^.

#### Motif enrichment analysis (macrophages)

Motif enrichment analysis was performed with R package monaLisa (v1.8) against HOCOMOCO v12 (in vivo subcollection) PWMs. Peaks within topics in the top and bottom 3% quantile of feature weights for MOFA factors of interest were considered for analysis. TFs with a false discovery rate (FDR) of 0.1% and log_2_ enrichment in the top or bottom 10% quantiles were considered significant.

#### Marker gene expression of T cell subsets (T cells)

Marker genes specific to each Th subset were taken from the state-specific gene signatures defined by Cano-Gamez and collaborators^52^ in their Supplementary Data 5 and used as gene sets for input to the ranking-based scoring approach AUCell.

Additional marker genes specific to the Th1 subset (Supplementary Fig. 1) were obtained by comparing Th1 subsets against naive T cell-derived Th0 cells from ref. ^52^ using DESeq2. Genes with adjusted *P* value <0.05 and log_2_ fold change >0 that were not differentially expressed in other Th subsets were used as Th1 signature for AUCell.

#### Differential accessibility and gene expression analyses (T cells, arrayed CRISPR screen)

Differentially expressed genes, differentially accessible peaks and differentially accessible TF motifs (chromVAR deviations) were identified using the Wilcoxon test as implemented in presto^95^. For T cells, we compared the cells that were stimulated with those that were restimulated, as well as each of the subsets within a stimulation to identify subset-specific results. Peaks with an adjusted *P* value <0.001 were considered significant. Stimulation-specific TFs were identified with a positive log fold change (logFC), an adjusted *P* value <0.001 and an area under the curve (AUC) of >0.9. Subset-specific TFs were selected to have logFC >0, adjusted *P* value <0.001 and AUC >0.7, and not be identified as stimulation specific. For the arrayed CRISPR screen, differential motif accessibility and gene expression analysis was performed within each line (CRISPRi/a) and day of differentiation, comparing cluster and gRNA annotated cell populations against all other cells within a day. Population-specific TFs were selected to have an adjusted *P* value <0.01 and AUC > 0.7, and genes to have an adjusted *P* value <0.01 and AUC >0.55.

#### Pseudotime inference (arrayed CRISPR screen)

First, a partition-based graph abstraction was generated using sc.tl.paga() on principal components of the transcriptomic data, with clusters defined by the Leiden algorithm. We initialized the layout for visualization by recalculating the graph layout with sc.tl.draw_graph(init_pos = ‘paga’). To perform diffusion pseudotime inference, we specified a root cell in the Leiden cluster with day 0 control cells, serving as a starting point for pseudotime calculation with sc.tl.dpt().

#### GSEA (macrophages, arrayed CRISPR screen)

For the macrophage experiment, gene set enrichment analysis (GSEA) was performed for Reactome database gene sets (v59). For every gene set, significance was evaluated via a parametric *t*-test, comparing weights of the foreground against a background set. For the arrayed CRISPR screen, GSEA was performed for differentially expressed genes for MSigDb C5 and WikiPathways gene sets using the R package clusterProfiler v4.4.4 function enricher() and enrichWP(), respectively. Enrichments with an adjusted *P* value <0.05 were considered significant.

#### GRN inference and TF prioritization (macrophages, arrayed CRISPR screen)

An eGRN was inferred using GRaNIE^37^ v1.5.3 with TFBS predictions based on the HOCOMOCO v12 (ref. ^94^) database (in vivo subset) that were generated using PWMScan (see ref. ^96^ for methodological details how these were produced). The single-cell data from all time points and stimulations was clustered using the smart local moving algorithm with a resolution of 1, giving rise to 31 clusters that have at least 25 cells. Mean RNA and ATAC values for each of these clusters as a pseudobulk were calculated, and input as ‘samples’ to create a eGRN following the GRaNIE single-cell vignette^97^. TF-peak connections with FDR <0.2 and peak-gene connections with FDR <0.1 were retained. TF motif connections were collapsed to the level of TFs to construct TF regulons.

#### GRN UMAP projection (arrayed CRISPR screen)

First, regulon activity was quantified per cell by AUCell. Second, Pearson correlations of regulon activity were computed for each pair of regulons (from the regulon-by-cell AUCell activity matrix), providing a regulon–regulon matrix. Next, principal component analysis was performed on the matrix and the top 20 PCs used as input for UMAP (uwot v0.1.14). To define the pseudotime point of their highest activity, the sum of pseudotime values for each cell multiplied by its regulon activity score were computed and divided by the sum of the regulon activity score. In turn, to assign a day to each regulon, the highest mean regulon activity was determined. Last, to define regulon importance in the network, PageRank centrality of each regulon was computed.

#### Residual analysis (macrophages)

To identify discrepancy and concordance between TF motif accessibility and regulon activity scores, values were rank min–max normalized and the difference between the two was computed and is referred to as the residual value.

#### GWAS integration (macrophages)

##### LDSC

To integrate GWAS with our eGRN, we performed stratified LDSC^44^ (s-LDSC v1.0.1). As a sanity check, we first tested whether enrichment for all open regions in all macrophages returned macrophage-related traits, such as blood cell traits. Once that was clear, we added these general cell-type regions as background, on top of the default baseline model that includes genic regions, enhancer regions and conserved regions. We tested enriched heritability for all peaks that were part of the STAT1/STAT2/IRF9 eGRN, all peaks in the eGRN and all peaks in M1 and M2 cells only. We included 8,890 traits from the UK Biobank, FinnGen and several GWAS repositories as included in the 22.10 release of Open Targets Genetics^98^. SNP heritability (h2) was calculated with defaults from the LDSC codebase. We tested S-LDSC only if h2 was >0.05, resulting in a list of 1,230 traits. We corrected the enrichment *P* values for the number of peak sets tested in each trait.

##### Gene modules and SNP overlap

For those traits that were enriched for heritability (adjusted *P* value <0.1), the SNPs associated to the particular trait (suggestive GWAS *P* value <5 × 10^−6^) were intersected with the peaks in the STAT1/STAT2/IRF9, and full eGRNs (Supplementary Tables 7 and 8). We selected the genes connected (in the STAT1/STAT2/IRF9 eGRN) to the peaks that overlapped a SNP to obtain disease gene modules for CD, IBD and eosinophil counts.

### Ethics declarations

hiPS cells used for macrophage generation were derived from PBMCs with institutional review board approval (Stanford University, reference numbers 29904 and 30064). The use of hiPS cells and primary human T cells from healthy donor buffy coats was approved by the EMBL Research Ethics Committee.

### Reporting summary

Further information on research design is available in the Nature Portfolio Reporting Summary linked to this article.

## Online content

Any methods, additional references, Nature Portfolio reporting summaries, source data, extended data, supplementary information, acknowledgements, peer review information; details of author contributions and competing interests; and statements of data and code availability are available at 10.1038/s41592-025-02700-8.

## Supplementary information

Supplementary Information Supplementary Tables 1–19 and Figs. 1–4.

Reporting Summary

Supplementary Tables 1–19 except 11, 16 and 17 Supplementary Tables 1–19 except 11, 16 and 17.

Supplementary Table 11 Supplementary Table 11.

Supplementary Table 16 Supplementary Table 16.

Supplementary Table 17 Supplementary Table 17.

## Data Availability

Data related to the species mixing experiment are available at GEO with accession number GSE253165. The macrophage polarization, T cell differentiation and arrayed CRISPR screen data are available on the European Genome and Phenome Archive under dataset IDs EGAD50000001206, EGAD50000001204 and EGAD50000001205, respectively.

## Code availability

A Snakemake preprocessing pipeline for SUM-seq data is available in a public Git repository^14^. Analysis source code and files are available at https://git.embl.de/grp-zaugg/sum-seq_analysis.
