# Supplementary material for: Single-cell ultra-high-throughput multiplexed chromatin and RNA profiling reveals gene regulatory dynamics
Source: Nat Methods. 2025 May 26;22(6):1213–25. doi: 10.1038/s41592-025-02700-8 (PMC12165849; doi:10.1038/s41592-025-02700-8)
Supplement: Supplementary file 1 — Supplementary Tables 1–19 and Figs. 1–4. [file 41592_2025_2700_MOESM1_ESM.pdf]

nature methods

Supplementary information https://doi.org/10.1038/s41592-025-02700-8

Single-cell ultra-high-throughput multiplexed chromatin and RNA profiling reveals gene regulatory dynamics

In the format provided by the authors and unedited

Supplementary Table 1: SUM-seq oligonucleotide sequences
Supplementary Table 2: M1 and M2 marker genes used for determining M1 and M2 scores

Column names:
Polarisation = *M1 or M2 polarisation*
Gene = *marker gene name*

Supplementary Table 3: Gene set enrichment analysis (GSEA) for M1 and M2 early, sustained and late response factors

Each tab contains the GSEA for M1 or M2. Column names:
Reactome pathway = pathways of the Reactome database
Factor = adjusted p-values for GSEA of gene sets associated with selected MOFA factors (associated with early, sustained and late M1 and M2 responses)

Supplementary Table 4: Top variable transcription factor (TF) motifs identified by ChromVAR and and motif enrichment in M1 and M2 MOFA (Multi-Omics Factor Analysis) factors

Tabs a) and b) contain the top 10% most variable TF motifs in M1/M2 polarization and filtered by those enriched in early, sustained and late response factors

Tabs c) and d) -log10 adjusted p values for motif enrichment analysis on cistopics associated with M1/M2-polarisation relevant latent factors. Columns are cistopics and rows are motifs.

Tabs e) and f) log 2 enrichment for motif enrichment analysis on cistopics associated with M1/M2-polarisation relevant latent factors. Columns are cistopics and rows are motifs

Supplementary Table 5: Gene regulatory network (GRN) connections for macrophages, filtered for TF-peak FDR =< 0.2 and peak-gene FDR =< 0.1
Column names:
TF.ID, TF.name, TF.ENSEMBL = *Transcription factor motif, name and ENSEMBL ID*
peak.ID = *Location of the peak (chromosome:start-end)*
peak.mean, peak.median = *Mean and median accessibility of peak*
peak.CV = *Coefficient of variation of accessibility of peak*
peak.annotation = *Type of peak (e.g. intronic, promoter, intergenic)*
peak.nearestGene.chr, peak.nearestGene.start, peak.nearestGene.end = *Location of the nearest gene from this peak (chromosome, start, end)*

peak.nearestGene.length, peak.nearestGene.strand, peak.nearestGene.distanceToTSS = *Length, strand and distance to transcription start site of nearest gene from this peak*

peak.nearestGene.name, peak.nearestGene.ENSEMBL, peak.nearestGene.symbol = *full name, ENSEMBL ID and name of nearest gene from this peak*

peak.GC.perc = *Percentage of GC content in peak*

TF_peak.r, TF_peak.r_bin = *Correlation coefficient and bin between TF and peak*

TF_peak.fdr, TF_peak.fdr_direction = *False discovery rate and direction of effect of correlation between TF and peak*

TF_peak.connectionType = *Method used to calculate TF activity*

peak_gene.distance = *Distance between peak and target gene*

peak_gene.p_raw, peak_gene.p_adj = *Raw and adjusted p-value for correlation between peak and target gene*

gene.ENSEMBL, gene.name = *ENSEMBL ID and name of target gene*

gene.type = *Type of target gene (e.g. protein coding, lncRNA)*
gene.mean, gene.median = *Mean and median expression of target gene*
gene.CV = *Coefficient of variation of expression of target gene*
gene.chr, gene.start, gene.end = *Location of target gene (chromosome, start, end)*
gene.strand = *Strand of target gene*
TF_gene.r, TF_gene.p_raw = *Raw p-value and correlation coefficient between TF and target gene*

Supplementary Table 6: STAT1 regulons from the GRN
Columns: STAT1 motif (first column), target gene name (second column)

Supplementary Table 7: Disease SNP overlap with all GRN regulons
Column names:
SNP = *Single nucleotide polymorphism rs ID*
peak = *GRN peak that overlaps with the SNP*
SNP.Z = *GWAS Z-score for the SNP corresponding to A1 allele*
SNP.A1, SNP.A2 = *Reference (A1) and alternative (A2) SNP alleles*
TF.name, TF.ENSEMBL = *Name and ENSEMBL ID for TF linked to peak*
gene.name, gene.ENSEMBL = *Name and ENSEMBL ID for gene linked to peak*
gene.chr, gene.start, gene.end = *Location of target gene (chromosome, start, end)*
peak_gene.p_adj = *Adjusted p-value for correlation between peak and target gene*
peak_gene.r = *Correlation coefficient between peak and gene*
disease = *GWAS trait where the SNP was identified (format: disease_studyID)*

Supplementary Table 8: Disease SNP overlap with STAT1/STAT2/IRF9 regulon
Column names as in Supplementary Table 7

Supplementary Table 9: Expression, protein, splicing quantitative trait loci (e-, p-, sQTLs) for rs4810485, collated by Open Targets Genetics
The tabs show results for eQTLs, pQTLs and sQTLs.
Columns indicate the dataset, rows the genes, values the significance as displayed on Open Targets website.

Supplementary Table 10: Differential gene expression calculated per stimulation condition (stimulated or restimulated) and per subset (results with p-value < 0.1).
Column names:
feature = *Gene*
group = *stimulation condition (5d = stimulated, 5d4h = restimulated), or T cell subset (IFNb, iTreg, Th0, Th1, Th17, Th2) in combination with the stimulation condition (_5d or _5d4h)*
avgExpr = *mean deviation of the TF in that set of cells*
logFC = *log fold change between observations in group vs out*
statistic = *Wilcoxon rank sum U statistic*
auc = *area under the receiver operator curve*
pval = *Wilcoxon p-value*
padj = *Benjamini-Hochberg adjusted p-value*
pct_in = *Percent of observations in the group with non-zero feature value*
pct_out = *Percent of observations out of the group with non-zero feature value*

Supplementary Table 11: Differential peak accessibility calculated per stimulation condition (stimulated or restimulated) and per subset (results with p-value < 0.1).
Column names:
feature = *ATAC peak*
all others as in Supplementary Table 10

Supplementary Table 12: Differential chromVAR deviations per transcription factor (TF) motif calculated per stimulation condition (stimulated or restimulated) and per subset.

Column names:

feature = *TF motif*

all others as in Supplementary Table 10

Supplementary Table 13: Composition of the arrayed CRISRPi/a screen used for SUM-seq of lineage factors in hiPSC-derived embryoid bodies and monolayer differentiation.

sgRNA_ID = *gRNA identification in the focused library*

sgRNA_ID_source_library = *original gRNA id from the Dolcetto (Sanson et al.) and the hCRISPRi-v2* (Horlbeck et al.) *libraries*

sgRNA_sequence [5'-3'] = *20 nt crRNA protospacer sequence adjacent to the PAM motif at the target site*

Oligonucleotide_sequence [5'-3'] = *oligonucleotide sequences in the pool for cloning into the modified CROP-seq-puroR-T2A-tagBFP or the pXPR502 lentivectors*

Supplementary Table 14: Gene regulatory network (GRN) connections for CRISPRa arrayed screen, filtered for TF-peak FDR =< 0.2 and peak-gene FDR =< 0.1

Column names:

TF.ID, TF.name, TF.ENSEMBL = *Transcription factor motif, name and ENSEMBL ID*

peak.ID = *Location of the peak (chromosome:start-end)*

peak.mean, peak.median = *Mean and median accessibility of peak*

peak.CV = *Coefficient of variation of accessibility of peak*

peak.annotation = *Type of peak (e.g. intronic, promoter, intergenic)*

peak.nearestGene.chr, peak.nearestGene.start, peak.nearestGene.end = *Location of the nearest gene from this peak (chromosome, start, end)*

peak.nearestGene.length, peak.nearestGene.strand, peak.nearestGene.distanceToTSS = *Length, strand and distance to transcription start site of nearest gene from this peak*

peak.nearestGene.name, peak.nearestGene.ENSEMBL, peak.nearestGene.symbol = *full name, ENSEMBL ID and name of nearest gene from this peak*

peak.GC.perc = *Percentage of GC content in peak*

TF_peak.r, TF_peak.r_bin = *Correlation coefficient and bin between TF and peak*

TF_peak.fdr, TF_peak.fdr_direction = *False discovery rate and direction of effect of correlation between TF and peak*

TF_peak.connectionType = *Method used to calculate TF activity*

peak_gene.distance = *Distance between peak and target gene*

peak_gene.p_raw, peak_gene.p_adj = *Raw and adjusted p-value for correlation between peak and target gene*

gene.ENSEMBL, gene.name = *ENSEMBL ID and name of target gene*

gene.type = *Type of target gene (e.g. protein coding, lncRNA)*

gene.mean, gene.median = *Mean and median expression of target gene*

gene.CV = *Coefficient of variation of expression of target gene*

gene.chr, gene.start, gene.end = *Location of target gene (chromosome, start, end)*

gene.strand = *Strand of target gene*

TF_gene.r, TF_gene.p_raw = *Raw p-value and correlation coefficient between TF and target gene*

Supplementary Table 15: Gene regulatory network (GRN) connections for CRISPRi arrayed screen, filtered for TF-peak FDR =< 0.2 and peak-gene FDR =< 0.1

Column names:

TF.ID, TF.name, TF.ENSEMBL = *Transcription factor motif, name and ENSEMBL ID*

peak.ID = *Location of the peak (chromosome:start-end)*

peak.mean, peak.median = *Mean and median accessibility of peak*

peak.CV = *Coefficient of variation of accessibility of peak*

peak.annotation = *Type of peak (e.g. intronic, promoter, intergenic)*

peak.nearestGene.chr, peak.nearestGene.start, peak.nearestGene.end = *Location of the nearest gene from this peak (chromosome, start, end)*

peak.nearestGene.length, peak.nearestGene.strand, peak.nearestGene.distanceToTSS = *Length, strand and distance to transcription start site of nearest gene from this peak*

peak.nearestGene.name, peak.nearestGene.ENSEMBL, peak.nearestGene.symbol = *full name, ENSEMBL ID and name of nearest gene from this peak*

peak.GC.perc = *Percentage of GC content in peak*

TF_peak.r, TF_peak.r_bin = *Correlation coefficient and bin between TF and peak*

TF_peak.fdr, TF_peak.fdr_direction = *False discovery rate and direction of effect of correlation between TF and peak*

TF_peak.connectionType = *Method used to calculate TF activity*

peak_gene.distance = *Distance between peak and target gene*

peak_gene.p_raw, peak_gene.p_adj = *Raw and adjusted p-value for correlation between peak and target gene*

gene.ENSEMBL, gene.name = *ENSEMBL ID and name of target gene*

gene.type = *Type of target gene (e.g. protein coding, lncRNA)*

gene.mean, gene.median = *Mean and median expression of target gene*

gene.CV = *Coefficient of variation of expression of target gene*

gene.chr, gene.start, gene.end = *Location of target gene (chromosome, start, end)*

gene.strand = *Strand of target gene*

TF_gene.r, TF_gene.p_raw = *Raw p-value and correlation coefficient between TF and target gene*

Supplementary Table 16: Differential gene expression within each DIV for CRISPRa cells grouped by the combination of cluster and perturbation target. Analysis compared each group against all other cells within a DIV.

Supplementary Table 17: Differential gene expression within each DIV for CRISPRi cells grouped by the combination of cluster and perturbation target. Analysis compared each group against all other cells within a DIV.

Supplementary Table 18: Differential motif accessibility within each DIV for CRISPRa cells grouped by the combination of cluster and perturbation target. Analysis compared each group against all other cells within a DIV.

Supplementary Table 19: Differential motif accessibility within each DIV for CRISPRi cells grouped by the combination of cluster and perturbation target. Analysis compared each group against all other cells within a DIV.

Supplementary Figure 1

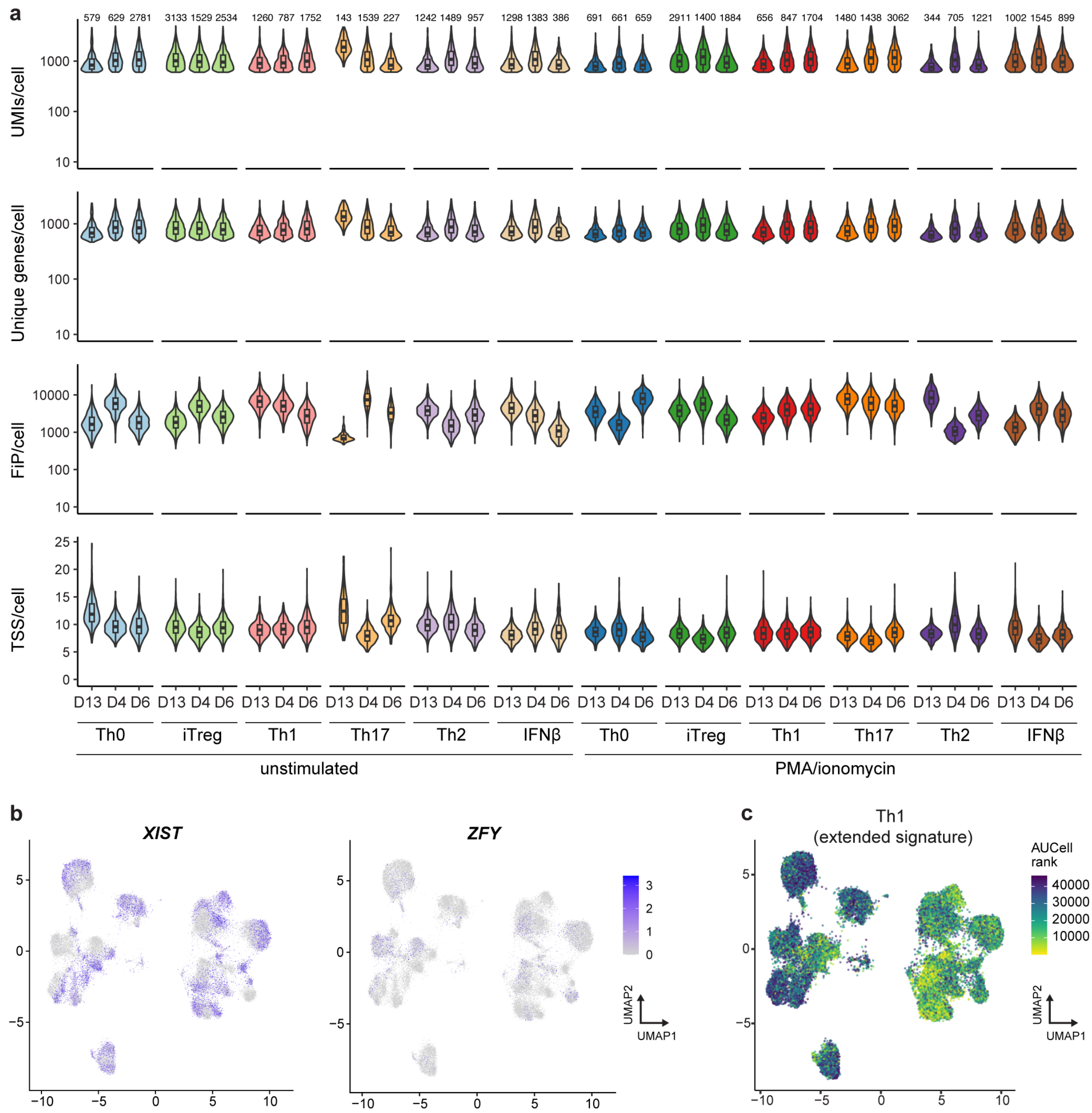

Supplementary Figure 1. a, Violin plots showing quality control metrics for RNA (UMIs per cell, genes per cell) and ATAC (FiP; Fragments in Peaks per cell, TSS enrichment per cell) for the T cell differentiation SUM-seq experiment across donors, subsets and stimulations. Center line represents median; lower and upper hinges represent the 25th and 75th quartiles respectively. The whiskers represent values that fall within 1.5 times the interquartile range between the 25th and 75th percentiles. Number of cells indicated above the violin plots. **b**, Weighted nearest neighbor (WNN) UMAP projection of integrated data from T cell SUM-seq experiment showing the RNA expression of XIST (only expressed in females) and ZFY (only expressed in males), **c**, Weighted nearest neighbor (WNN) UMAP projection of integrated data from T cell SUM-seq experiment. Weighted Nearest Neighbor (WNN) UMAP projection of integrated SUM-seq data from Th subsets. Cells are colored according to their “expanded” Th1 AUCell scores, generated using a larger number of Th1 markers than in Data Figure 4 (Methods) to further validate the identity of Th1 cells.

Supplementary Figure 2

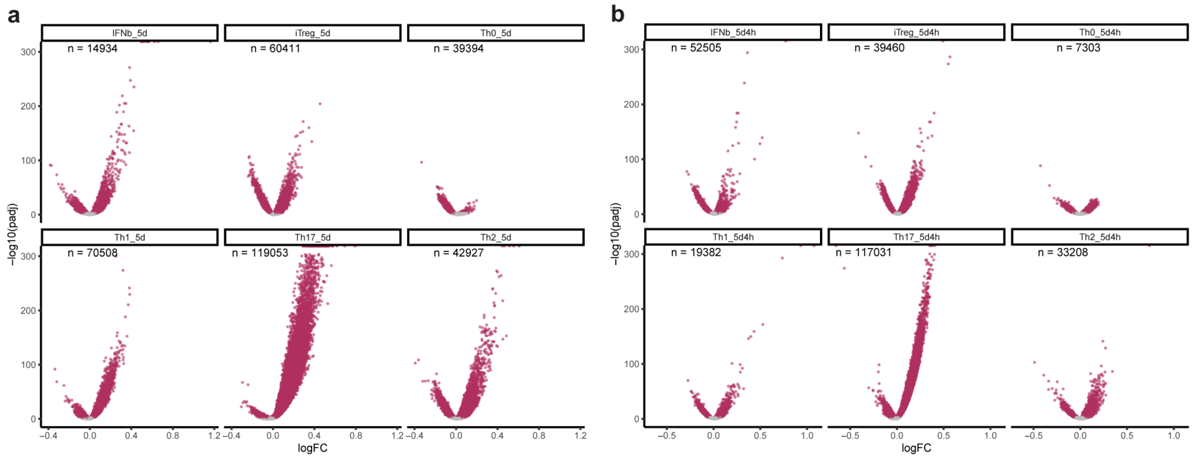

Supplementary Figure 2. Volcano plots depicting differentially accessible peaks in T cell subset upon stimulation (**a**) and restimulation (**b**). Significantly differentially accessible peaks ($p < 0.001$) are colored and the number is printed on each Volcano plot (n). x-axis is log2(fold change) values and y-axis -log10(FDR corrected p-values) (Wilcoxon rank-sum test). The number of cells used to calculate values are: a) IFNb_5d (3067), iTreg_5d (7196), Th0_5d (3989), Th1_5d (3799), Th17_5d (1909), Th2_5d (3688). b) IFNb_5d4h (3446), iTreg_5d4h (6195), Th0_5d4h (2011), Th1_5d4h (3207), Th17_5d4h (5980), Th2_5d4h (2270)

Supplementary Figure 3

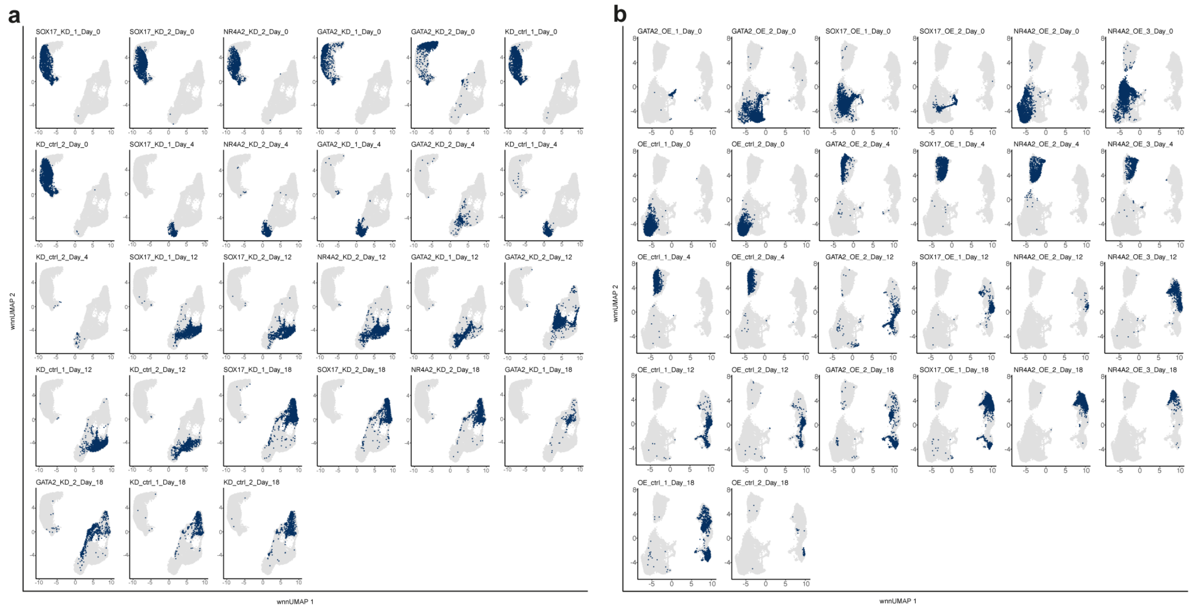

Supplementary Figure 3. WNN-UMAPs from the arrayed CRISPRi (**a**) and CRISPRa (**b**) screens split for each sample and timepoint.

Supplementary Figure 4

a

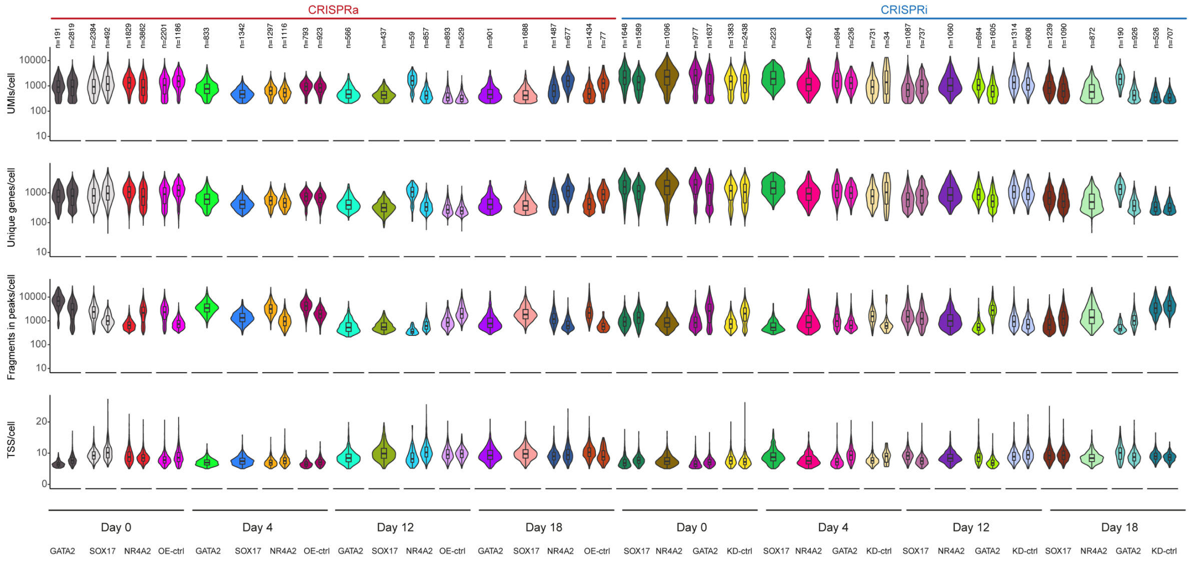

Supplementary Figure 4. Distribution of quality control metrics of RNA-seq (UMIs, top row and unique genes per cell, second row) and ATAC-seq (fragments in peaks, third row and TSS enrichment per cell, bottom row) in the arrayed CRISRPi/a screens across perturbations and differentiation times. Center line represents median; lower and upper hinges represent the 25th and 75th quartiles respectively. The whiskers represent values that fall within 1.5 times the interquartile range between the 25th and 75th percentiles. n indicates the number of cells per condition and is shown above the violin plots.
