# Supplementary material for: Single-cell ultra-high-throughput multiplexed chromatin and RNA profiling reveals gene regulatory dynamics
Source: Nat Methods. 2025 May 26;22(6):1213–25. doi: 10.1038/s41592-025-02700-8 (PMC12165849; doi:10.1038/s41592-025-02700-8)
Supplement: Supplementary file 2 — Reporting Summary [file 41592_2025_2700_MOESM2_ESM.pdf]

Corresponding author(s): Judith Zaugg, Mikael Marttinen, Min Noh

Last updated by author(s): Feb 5, 2024

Reporting Summary

Nature Portfolio wishes to improve the reproducibility of the work that we publish. This form provides structure for consistency and transparency in reporting. For further information on Nature Portfolio policies, see our Editorial Policies and the Editorial Policy Checklist.

Statistics

For all statistical analyses, confirm that the following items are present in the figure legend, table legend, main text, or Methods section.

n/a	Confirmed	
☐	☒	The exact sample size (n) for each experimental group/condition, given as a discrete number and unit of measurement
☐	☒	A statement on whether measurements were taken from distinct samples or whether the same sample was measured repeatedly
☐	☒	The statistical test(s) used AND whether they are one- or two-sided<br>*Only common tests should be described solely by name; describe more complex techniques in the Methods section.*
☒	☐	A description of all covariates tested
☐	☒	A description of any assumptions or corrections, such as tests of normality and adjustment for multiple comparisons
☐	☒	A full description of the statistical parameters including central tendency (e.g. means) or other basic estimates (e.g. regression coefficient) AND variation (e.g. standard deviation) or associated estimates of uncertainty (e.g. confidence intervals)
☐	☒	For null hypothesis testing, the test statistic (e.g. F, t, r) with confidence intervals, effect sizes, degrees of freedom and P value noted<br>*Give P values as exact values whenever suitable.*
☒	☐	For Bayesian analysis, information on the choice of priors and Markov chain Monte Carlo settings
☒	☐	For hierarchical and complex designs, identification of the appropriate level for tests and full reporting of outcomes
☐	☒	Estimates of effect sizes (e.g. Cohen's d, Pearson's r), indicating how they were calculated

Our web collection on statistics for biologists contains articles on many of the points above.

Software and code

Policy information about availability of computer code

Data collection	Not software was used for data collection
Data analysis	Data analyses were performed in R v4.2.2 and Python v3.9.13. The pipeline used to pre-process the data is accessible from https://git.embl.de/grp-zaugg/SUMseq, it includes bcl-convert (v4.0.3), chromap (v0.2.3), ArchR (v1.02), bcl2fastq (v2.20.0), Je (v2.0.RC), STARsolo (v2.7.11a), EmptyDrops (v1.16). All code used to perform data analysis are available from https://git.embl.de/grp-zaugg/SUMseq_analyses. Tools and databases used are MACS2 (v2.2.9.1), AUCell (v1.20.2), HOCOMOCO (v12), MOFA (v1.6.0), cisTopic (v0.3), Reactome (v59), monaLisa (v1.8), GRaNIE (v1.5.3), LDSC (v1.01), dbSNP (v155).

For manuscripts utilizing custom algorithms or software that are central to the research but not yet described in published literature, software must be made available to editors and reviewers. We strongly encourage code deposition in a community repository (e.g. GitHub). See the Nature Portfolio guidelines for submitting code & software for further information.

Data

Policy information about availability of data

All manuscripts must include a data availability statement. This statement should provide the following information, where applicable:

- Accession codes, unique identifiers, or web links for publicly available datasets
- A description of any restrictions on data availability
- For clinical datasets or third party data, please ensure that the statement adheres to our policy

Data related to the species mixing experiment is available at GEO with accession number GSE253165. The macrophage polarisation, T cell differentiation, and arrayed CRISPR screen data are available on the European Genome and Phenome Archive under dataset IDs EGAD50000001206, EGAD50000001204, and EGAD50000001205, respectively.

Human research participants

Policy information about studies involving human research participants and Sex and Gender in Research.

Reporting on sex and gender	We describe the sex of the cell lines in the text.
Population characteristics	N/A
Recruitment	N/A
Ethics oversight	hiPSCs used for macrophage generation were derived from peripheral blood mononuclear cells with institutional review board approval (Stanford University, reference numbers 29904, 30064). The use of hiPSCs was approved by the EMBL Research Ethics Committee.

Note that full information on the approval of the study protocol must also be provided in the manuscript.

Field-specific reporting

Please select the one below that is the best fit for your research. If you are not sure, read the appropriate sections before making your selection.

☒ Life sciences ☐ Behavioural & social sciences ☐ Ecological, evolutionary & environmental sciences

For a reference copy of the document with all sections, see nature.com/documents/nr-reporting-summary-flat.pdf

Life sciences study design

All studies must disclose on these points even when the disclosure is negative.

Sample size	Sample size was determined according to the standards of the field with two-three biological replicates for the single cell experiments.r
Data exclusions	No dataset was excluded. The single cell data sets were filtered to exclude droplets that likely contain no cells and only ambient material. For this, cells were filtered using EmptyDrops (v.1.16) or based on the inflection point of cell features (UMI per cell (snRNA-seq); fragments per cell and TSS enrichment score per cell (snATAC-seq) as described in the methods section.
Replication	We replicated the technology across four different systems.
Randomization	All experiments were done at the same time, there was no randomization necessary
Blinding	All experiments were done at the same time, and sequencing was performed at the same time, the experimenter had no influence on the outcome therefore blinding was not necessary.

Reporting for specific materials, systems and methods

We require information from authors about some types of materials, experimental systems and methods used in many studies. Here, indicate whether each material, system or method listed is relevant to your study. If you are not sure if a list item applies to your research, read the appropriate section before selecting a response.

Materials & experimental systems

n/a	Involved in the study
☐	☒ Antibodies
☐	☒ Eukaryotic cell lines
☒	☐ Palaeontology and archaeology
☒	☐ Animals and other organisms
☒	☐ Clinical data
☒	☐ Dual use research of concern

Methods

n/a	Involved in the study
☒	☐ ChIP-seq
☒	☐ Flow cytometry
☒	☐ MRI-based neuroimaging

Antibodies

Antibodies used: anti-IFN-γ (R&D systems, MAB285-SP, Clone # 25718)

Validation: Biological Validation, Immunocytochemistry, Intracellular Staining by Flow Cytometry, Neutralization

Eukaryotic cell lines

Policy information about cell lines and Sex and Gender in Research

Cell line source(s):
NIH-3T3 (DSMZ, ACC 59)
HEK293T (ATCC)
K562 (DSMZ, ACC 10)
human induced pluripotent stem cells (CESCG-295, male donor, peripheral blood mononuclear cell-derived; provider: Dr. Michael Snyder, Stanford University; approved Material Transfer agreement)

Authentication: none of the cell lines were authenticated

Mycoplasma contamination: cells were not tested for mycoplasma contamination

Commonly misidentified lines (See ICLAC register): No commonly missidentified lines were used
